# Hydroxychloroquine attenuates neuroinflammation following traumatic brain injury by regulating the TLR4/NF-κB signaling pathway

**DOI:** 10.1186/s12974-022-02430-0

**Published:** 2022-03-28

**Authors:** Jian Hu, Xue Wang, Xiongjian Chen, Yani Fang, Kun Chen, Wenshuo Peng, Zhengyi Wang, Kaiming Guo, Xianxi Tan, Fei Liang, Li Lin, Ye Xiong

**Affiliations:** 1 The First Affiliated Hospital of Wenzhou Medical University, Wenzhou, 325000 China; 2 School of Pharmaceutical Sciences, Wenzhou Medical University, University-town, Wenzhou, 325035 China; 3 Research Units of Clinical Translation of Cell Growth Factors and Diseases Research, Chinese Academy of Medical Science, Beijing, 100730 China

**Keywords:** Hydroxychloroquine, TBI, Neuroinflammation, Microglia activation, TLR4/NF-κB

## Abstract

**Background:**

After traumatic brain injury (TBI), an acute, robust inflammatory cascade occurs that is characterized by the activation of resident cells such as microglia, the migration and recruitment of peripheral immune cells and the release of inflammatory mediators that induce secondary cell death and impede neurological recovery. In addition, neuroinflammation can alter blood–brain barrier (BBB) permeability. Controlling inflammatory responses is considered a promising therapeutic approach for TBI. Hydroxychloroquine (HCQ) has already been used clinically for decades, and it is still widely used to treat various autoimmune diseases. However, the effects of HCQ on inflammation and the potential mechanism after TBI remain to be defined. The aim of the current study was to elucidate whether HCQ could improve the neurological recovery of mice post-TBI by inhibiting the inflammatory response via the TLR4/NF-κB signaling pathway.

**Methods:**

C57BL/6 mice were subjected to controlled cortical impact (CCI) and randomly divided into groups that received intraperitoneal HCQ or vehicle daily after TBI. TAK-242 (3.0 mg/kg), an exogenous TLR4 antagonist, was injected intraperitoneally 1 h before TBI. Behavioral assessments were performed on days 1 and 3 post-TBI, and the gene expression levels of inflammatory cytokines were analyzed by qRT-PCR. The presence of infiltrated immune cells was examined by flow cytometry and immunostaining. In addition, BBB permeability, tight junction expression and brain edema were investigated.

**Results:**

HCQ administration significantly ameliorated TBI-induced neurological deficits. HCQ alleviated neuroinflammation, the activation and accumulation of microglia and immune cell infiltration in the brain, attenuated BBB disruption and brain edema, and upregulated tight junction expression. Combined administration of HCQ and TAK-242 did not enhance the neuroprotective effects of HCQ.

**Conclusions:**

HCQ reduced proinflammatory cytokine expression, and the underlying mechanism may involve suppressing the TLR4/NF-κB signaling pathway, suggesting that HCQ is a potential therapeutic agent for TBI treatment.

**Supplementary Information:**

The online version contains supplementary material available at 10.1186/s12974-022-02430-0.

## Background

Traumatic brain injury (TBI) is a major health problem worldwide that is characterized by extensive neurologic disability and mortality [1, 2]. TBI is a multimodal complex disease process and not a single pathophysiological event [3]. After TBI, direct tissue loss and cell death lead to primary damage; subsequently, oxidative stress, neuroinflammation, blood–brain barrier (BBB) disruption and other factors contribute to secondary brain injury. At present, no standardized therapies are currently available to cure neurological deficits of TBI patients [4, 5]. TBI induces cellular damage, which in turn leads to the rapid release of damage-associated molecular patterns (DAMPs) that stimulate resident cells to release cytokines and chemokines [6]. The release and production of cytokines and chemokines contribute to the resulting inflammatory response [7, 8]. Inflammation contributes to early brain injury and is crucial to recovery after TBI. Moreover, exorbitant inflammation further damages BBB integrity and facilitates the invasion of even more peripheral immune cells [9, 10]. In recent years, the regulation of posttraumatic neuroinflammation has been a concern of researchers, and alleviating the inflammatory response has been reported to exert neuroprotective effects in a TBI model [11–13].

Microglia are the main immune cells of the brain, and respond to cerebral injury by becoming activated. After TBI, a robust inflammatory response occurs acutely, which is characterized by the activation of resident cells, such as microglia, the migration and recruitment of peripheral immune cells, and the release of inflammatory mediators [14]. Microglial activation results in inflammatory activation and triggers the release of several proinflammatory cytokines, such as TNF-α, inducible nitric oxide synthase (iNOS), and IL-1β, in the central nervous system (CNS), which play major roles in secondary damage after TBI [15, 16]. Inflammation is involved in the pathogenesis of TBI, contributes to early brain injury and is crucial to recovery, and alleviating the inflammatory response can ameliorate brain injury after TBI [11, 12, 17]. In addition, microglial activation can stimulate the synthesis of nitric oxide, which leads to BBB disruption and promotes increased release of inflammatory mediators, ultimately causing secondary brain injury. Although moderate immune cell activation is essential for eliminating cellular debris and danger signals, excessive and uncontrolled inflammation can exacerbate neurological impairment. In addition, excessive inflammation can further compromise BBB integrity, facilitating the invasion of additional peripheral immune cells (e.g., macrophages and neutrophils). BBB dysfunction is a significant posttraumatic event and is accompanied by disrupted tight junctions and increased paracellular permeability. Accumulating evidence indicates that perivascular microglia, especially M1-like microglia, contribute to BBB breakdown and that TNF-α secreted by M1 microglia leads to necroptosis in the endothelium, which is a key component of the BBB [18]. Toll-like receptor 4 (TLR4), which is a member of the TLR family, is an important transmembrane receptor involved in inflammatory processes [10]. The protein high-mobility group box 1 (HMGB1) is an important mediator of late inflammatory responses and activates TLR4 to further trigger downstream signaling pathways, such as the nuclear factor (NF)-κB pathway. NF-κB is activated by the degradation of IκBα, and the nuclear translocation of NF-κB triggers the cascade amplification of inflammatory responses, inducing secondary damage after TBI [19]. Therefore, suppressing TLR4/NF-κB-mediated TBI-induced microglial activation and modulating the subsequent neuroinflammatory response to an appropriate level have been demonstrated to facilitate recovery in TBI patients [20].

Chloroquine (CQ) and hydroxychloroquine (HCQ) are aminoquinoline drugs that were originally developed to treat malaria and have already been used clinically for decades [21]. In addition, although these are “old” drugs, they are still widely used to treat various autoimmune diseases, including systemic lupus erythematosus (SLE) [22, 23], rheumatoid arthritis (RA) [12] and other autoimmune diseases [24], due to their anti-inflammatory and immunomodulatory properties [25, 26]. The difference between HCQ and CQ is that HCQ has an additional hydroxyl group and reduced toxicity while retaining its efficacy. The safety of HCQ has been universally verified in clinical practice [27]. HCQ has potential neuroprotective effects, and studies have shown that after long-term HCQ treatment, a variety of tissues, including brain tissue, accumulate sufficient drug levels to induce beneficial effects [28]. Based on the powerful immunomodulatory effect of HCQ, it has also been used to treat some nervous system diseases. In a multiple sclerosis (MS) animal model of autoimmune encephalomyelitis (EAE), HCQ was proven by Koch et al. to reduce microglial activation and the occurrence of MS disease [28]. Previous studies have revealed that HCQ has anti-inflammatory ability. HCQ/CQ could inhibit cathepsin and NLRP3 [25] and modulate immune players, such as toll-like receptors (TLR3, TLR4, TLR7, TLR8, TLR9), interferons (IFNs), mitogen-activated protein kinase (MAPK), chemokines, and generation of reactive oxygen species (ROS) [29, 30]. HCQ/CQ have shown protective effects in TBI [31]. Nevertheless, the anti-inflammatory mechanism of HCQ/CQ in the TBI has not been clearly explored, such as its effect on the TLR4/NF-κB pathway and the mechanism of inhibiting the activation of microglia. In addition, the influences of HCQ/CQ on BBB and immune infiltration after TBI have not been specifically reported. In this study, we investigated the effects of HCQ in an experimental TBI model. We hypothesized that HCQ would inhibit neuroinflammation through the TLR4/NF-κB signaling pathway and ameliorate BBB disruption, thereby improving neurological outcomes in early brain injury after TBI.

## Materials and methods

### Animals and controlled cortical impact model

Male C57BL/6 mice aged 6–8 weeks (20–25 g) were purchased from the Animal Center of the Chinese Academy of Sciences (Beijing, China), and were housed in cages maintained at 25 °C with 12 h/12 h light–dark schedule. The mice had free access to food and water. All animal care and use were approved by the Laboratory Animal Ethics Committee of Wenzhou Medical University.

A controlled cortical impact (CCI) model was performed to establish a TBI model using a device (RWD, Shenzhen, China) as we previously described [32]. In brief, mice were anesthetized by isoflurane, placed on a heating blanket to maintain body temperature at 37 ± 0.5 °C, and mounted in the stereotaxic frame. The injury was induced by a 3 mm impactor at a velocity of 4 m/s and 1.0 mm depth of penetration, in the left hemisphere of mice. Subsequently, the mice were placed into an incubator maintained at 37 °C to recover. The sham group mice underwent the same procedure without impacting.

### Animal experimental procedures

**Fig. 1:**
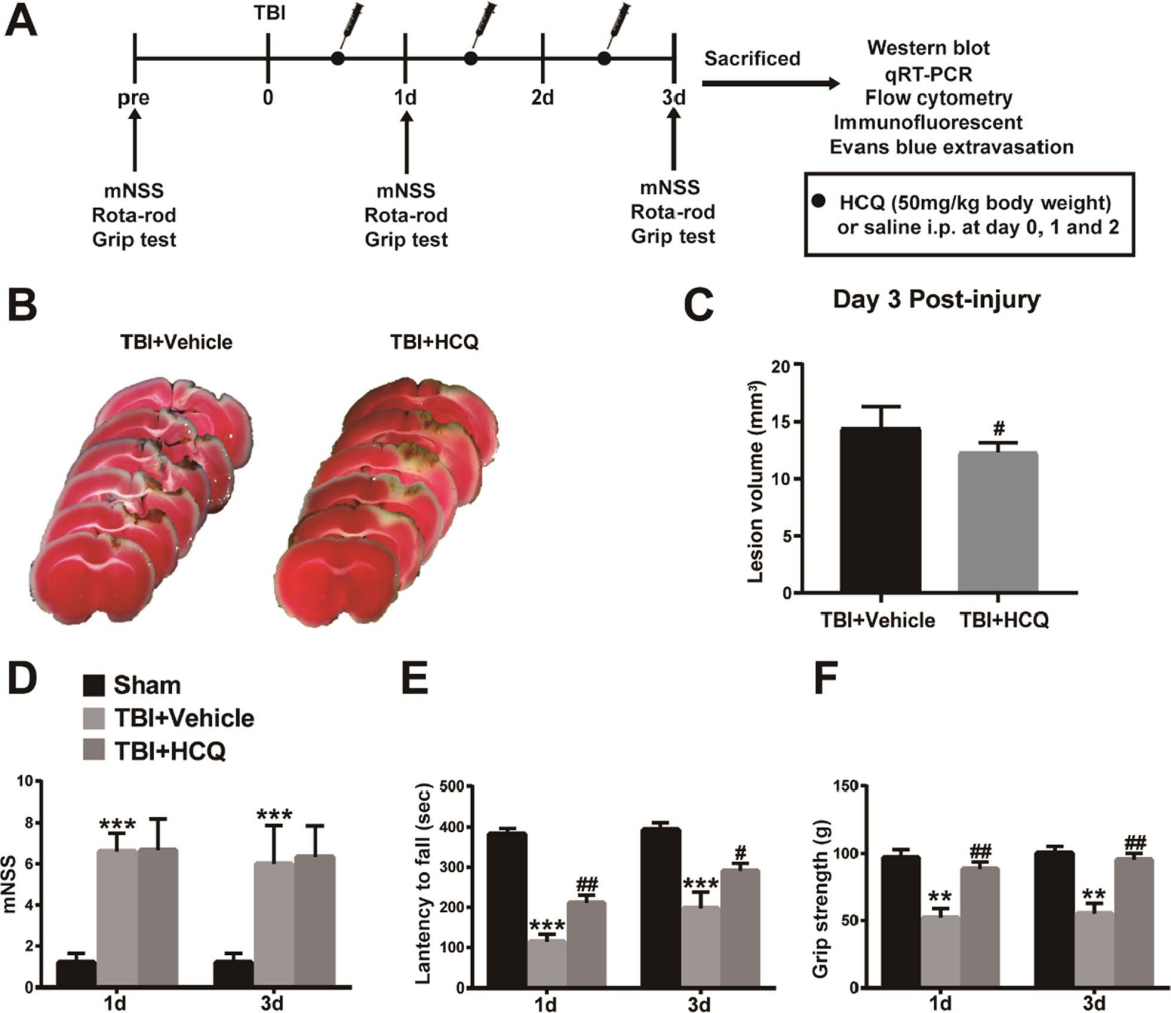
Effect of HCQ on cortical size and neurological function after TBI. **a** Schematic representation of the TBI model and experimental design. **b** Representative TTC staining of brain sections at 3 day post-TBI. **c** Quantitative analysis of the lesion volume in TTC-stained brain sections, *n* = 5 per group. The data are presented as the mean ± SD, ^#^*p* < 0.05 vs. TBI + Vehicle. **d**–**f** Neurological recovery was evaluated by mNSS, the rotarod test, and the grip strength at 1 and 3 day post-TBI. *n* = 8 per group. The data are presented as the mean ± SD. **p < 0.01, ***p < 0.001 vs. sham group, ^#^*p* < 0.05, ^##^*p* < 0.01 vs. TBI + Vehicle group

In the present study, the following three experiments were conducted:

In Experiment 1, the study design and protocol for the animal experiments are illustrated in Fig. 1a. Male mice were randomly divided into three groups: the sham group, TBI + vehicle group (saline was administered), and TBI + HCQ group. HCQ was dissolved in saline. HCQ (50 mg/kg) or vehicle was injected intraperitoneally immediately following TBI once each day until sacrifice. All animals received three consecutive days of the adaptation before TBI induction. Behavioral tests were performed before TBI and on days 1 and 3 post-TBI, and these assessments were scored in a blinded manner. The mice were sacrificed three days after TBI, and Evans blue (EB) extravasation (*n* = 5/group), flow cytometry (*n* = 5/group), immunofluorescence staining (*n* = 5/group), quantitative real-time polymerase chain reaction (qRT-PCR) (*n* = 5/group), and western blotting (*n* = 5/group) were performed.

In Experiment 2, mice were randomly divided into four groups: TBI + vehicle, TBI + TAK-242, and TBI + HCQ, TBI + TAK-242 + HCQ group. An exogenous TLR4 antagonist, TAK-242 (3.0 mg/kg), was injected intraperitoneally 1 h before TBI. The study design and protocol are illustrated in Fig. 8a. Neurological scores, behavioral tests, qRT-PCR (*n* = 5/group), and western blot (*n* = 5/group) were performed.

In Experiment 3, we explored the therapeutic window of HCQ treatment for TBI (Fig. 9a). A total of 20 mice were randomly divided into 4 groups: the TBI + Vehicle group, TBI + HCQ (0 h) group, TBI + HCQ (3 h) group, and TBI + HCQ (6 h) group. Rotarod tests and grip strength tests were performed on days 1 and 3 post-TBI, and brain water content was measured at 3 day post-TBI.

### Cells and culture conditions

Primary microglia culture was isolated as previously reported [33]. In brief, the cerebral cortices were separated from the brains of P1–P3 postnatal Sprague–Dawley rats, and the blood vessels and menings were removed. Then, the cortical tissue was minced with ophthalmic scissors and digested with 0.25% (w/v) trypsin (Gibco, Thermo Fisher Scientific, USA) for 30 min. Then the cells were dispersed by gently pipetting, and filtrated with 70 µm cell strainer (Fisherbrand, Sigma-Aldrich, USA) to obtain the mixed cortical cells. Cells pellets were resuspended in DMEM/F12 with 10% FBS and plated on 25 cm^2^ flasks. Culture media were changed every 3 days. On the 9th day, mixed glia was cultured with media consisting of 75% DMEM/F12 (10% FBS) and 25% fibroblast-conditioned media (F-CM). Primary microglia were isolated via mild trypsinization on the 13th day, and cultured for other experiments.

### CCK-8 assay

Cell viability was measured by Cell Counting Kit-8 (CCK-8) (Beyotime Biotechnology, Shanghai, China). Primary microglia were seeded on 96-well plates and treated with various concentrations of HCQ (1, 5, 10, and 20 μM). Cells treated with PBS (0.1%, v/v) served as vehicle control. After 24 h incubation, CCK-8 regent (1:10) was added to each well, and the plate was incubated at 37 °C for 4 h. Absorbance was measured at 450 nm using a SpectraMAX M3 microplate reader (Molecular Devices, Sunnyvale, CA, USA).

### Neurological score assessment

The modified neurological severity score (mNSS) is a composite of motor, sensory, balance and reflex tests and has been widely used in TBI studies [34]. The mNSS was graded on a scale of 0 to 12 (normal: score 0; maximal deficit: score 12). More severe impairment of normal function results in a higher score. The neurological score on days 1 and 3 following TBI was blindly assessed as described previously [35]. The test was performed in a double-blinded manner by examiners.

### Rotarod test

Rota-rod test was carried out to assess the limb motor coordination and balance of mice on the automated rota-rod (San Diego Instruments, San Diego, CA, USA), as shown in previous study [36]. Mice in each group were trained for three consecutive days before TBI induction. Then, on days 1, 3 post-injury, each mouse was placed on the accelerating automated rota-rod, which accelerated from 5 to 40 rpm/min within 5 min. The latency to fall for each mouse was recorded. Each mouse was tested three times with the same speed each day with an interval of 30 min between trials, and the average latency to falling was used for analysis. The test was performed in a double-blinded manner by examiners.

### Grip strength test

Grip strength test was performed to measure the forelimb muscles grip strength by a grid grip strength meter (San Diego Instruments). Each mouse was pulled backward by its tail until it released its grip [32, 36]. Each animal was trained before TBI induction and then tested three times on days 1 and 3 after TBI. The average tensile strength value was used for analysis.

### Lesion volume measurement

2,3,5-triphenyltetrazolium chloride (TTC) staining was performed to measure the volume of cortical lesions. In brief, at 72 h after TBI, animals were anesthetized with pentobarbital (100 mg/kg) via intraperitoneal injection then perfused with saline and euthanized, and their brains were removed. The brain tissue was sliced into six 1-mm-thick slices in the coronal plane and stained with a 2% solution of TTC for 20 min. Images of the stained sections were taken. Lesion area included the infarct part were measured with image analysis software (ImageJ, NIH) and the sum of lesion volume was calculated.

### BBB permeability and brain edema determination

Extravasation of evans blue (EB) was used to assess the BBB permeability. Brain water content and EB leakage are two common methods to assess brain edema and BBB disruption [36]. In brief, 72 h after mice were subjected to TBI, EB solution (4 ml/kg, 2% in saline, Sigma-Aldrich, USA) was administrated via the tail vein and circulated for 2 h. Under deep anesthesia, mice were sacrificed by cardiac perfusion with cold phosphate buffered saline (PBS) to sufficiently eliminate the intravascular-localized dye. Then, the left hemisphere was immediately removed and weighed. The samples were homogenized and incubated for 72 h at 60 °C. After incubation, the tissue samples were centrifuged (14,000 rpm, 20 min), and absorbance of the supematant was measured with spectrofluorophotometer at an excitation wavelength of 610 nm and an emission wavelength of 680 nm [37]. The EB concentration was quantified according to a linear standard curve, and was expressed as micrograms per gram of brain tissue.

Brain water content was measured according the wet weight-dry weight method, as we previously described [36]. Briefly, 72 h after mice were subjected to TBI, under deep anesthesia, mice were sacrificed. The left hemispheres were rapidly removed, and weighted immediately to get the wet weight (WW), and were dried in an oven at 80 °C for 72 h until a constant weight was achieved to obtain the dry weight (DW). The brain water content was calculated as: (WW-DW)/WW × 100%.

### Flow cytometry

To assess the effects of HCQ on cellular components in the impaired hemisphere, infiltrating immune cells, peripheral inflammation, single cells from the brain, blood, and spleen were prepared for flow cytometric analysis 3 days after TBI, as previously reported [38]. Briefly, mice were euthanized, and the spleen and blood were harvested before the mice were perfused with ice-cold phosphate-buffered saline (PBS). The ipsilateral hemispheres of the brains were carefully dissected. Single-cell suspensions of the spleen and blood were prepared as previously described [38]. The spleen was gently pushed through a cell strainer (40 µm, Fisherbrand), and then red blood cell lysis buffer (Solarbio, Beijin, China) was added to remove red blood cells from the spleen and blood. Single-cell brain suspensions were prepared by neural tissue dissociation kits (Miltenyi Biotech, Bergisch Gladbach, Germany) based on the protocol. Briefly, the brain tissue was dissected and minced into small pieces in cold Hank's balanced salt solution (HBSS). Then, the tissue was passed through a cell strainer (70 μm, Fisherbrand) and centrifuged at 300 *g* at 4 °C for 5 min, followed by digestion in enzyme mix 1 (Neural Tissue Dissociation Kit) for 15 min at 37 °C and then digestion in enzyme mix 2 (Neural Tissue Dissociation Kit) for 10 min at 37 °C. Subsequently, the cells were washed with HBSS, and single cells were isolated by passing the pellets through a 30-µm cell strainer, followed by centrifugation at 300 *g* at 4 °C for 5 min. Cell pellets were obtained from the spleen, blood and brain and then subjected to further staining with fluorescently labeled antibodies as follows: CD45-PE (Cat# 103105, 1:100, Biolegend, San Diego, CA, USA), CD11b-APC (Cat# 101211, 1:100, Biolegend), and Ly6G-FITC (Cat# 127625, 1:100, Biolegend) or CD45-APC (Cat# 103111, 1:100, Biolegend), CD3-PE (Cat#100205, 1:100, Biolegend), and CD19-FITC (Cat# 152403, 1:100, Biolegend). Appropriate isotype controls were prepared, and fluorochrome compensation was performed with single-stained OneComp eBeads (eBioscience, Thermo fisher scientific, USA). Cells were analyzed by a FACS Aria flow cytometer (BD Bioscience, CA, USA), and the data were analyzed by FlowJo software (Informer Technologies, USA).

### Western bolt

Western blot analysis was performed to detect possible mechanism of HCQ attenuates neuro-inflammation following traumatic brain injury. Briefly, brain tissue from the lesion boundary zone of the cerebral cortex and harvested cell pellets were homogenized in ice-cold RIPA lysis buffer (Sigma-Aldrich) with the addition of protease and phosphatase inhibitor cocktail (Beyotime Biotechnology) to obtain total protein. Protein concentrations were quantified by the BCA protein assay kit (Beyotime Biotech) according to manufacturer’s instructions. An equal amount of protein (60 μg) was suspended in loading buffer (denatured at 100 °C for 5 min) and were separated by 8–12% SDS–PAGE and transferred to a polyvinylidene fluoride membrane (Millipore, Billerica, MA, USA). The membranes were blocked in 5% BSA for 1 h at room temperature and then incubated with primary antibodies against anti-iNOS (sc-7271, 1:300, Santa Cruze, CA, USA), anti-TNF-α (sc-52746, 1:300, Santa Cruze), anti-TLR4 (sc-293072, 1:300, Santa Cruze), anti-NF-κB p65 (Cat# 8242S, 1:1000, Cell Signaling Technology, Beverly, MA, USA), anti-p-NF-κB p65 (Cat# 3033S, 1:1000, Cell Signaling Technology), anti-IκBα (Cat# 4814S, 1:1000, Cell Signaling Technology), anti-p-IκBα (Cat# 2859S, 1:1000, Cell Signaling Technology), anti-ZO-1 (Cat# 21773–1-AP, 1:1000, Proteintech Group, Wuhan, China), anti-claudin-5 (BS1069, 1:1000, Bioworld Technology, Nanjing, China), anti-Iba-l (ab178846, 1:1000, abcam, Cambridge, MA, USA) and β-actin (AP0060,1:5000, Bioworld Technology), at 4 °C overnight. The membranes were incubated with the species-appropriate horseradish peroxidase (HRP)-conjugated secondary antibodies (1:10,000, all from abcam) for 1 h at room temperature. Finally, the bands were visualized with a ChemiDoc XRS^+^ Imaging System (Bio-Rad, CA, USA) and quantified using Image Lab 5.0 software (Bio-Rad, CA, USA).

### Immunofluorescence staining

Immunofluorescence staining was performed at 3 day post-TBI and 4 h after cell treatment, as previously described [33]. For microglia staining, mice were perfused with PBS, brains were excised and incubated in 4% paraformaldehyde for 12 h and then in sucrose 30% until sunk, 30-μm-thick coronal slices of frozen brains were cut with a cryostat (CM 1100; Leica). For other immunofluorescence staining, paraffin Sections (5 µm thick) were deparaffinized and rehydrated, and antigen retrieval was performed. After blocking nonspecific binding with 5% bovine serum albumin (BSA) and 0.3% Triton X-100 at 37 °C for 1 h, the sections were incubated with primary antibodies: Anti-Iba-1(Cat# ctf4377, 1:500, Wakao Osaka, Japan), anti-CD16 (AF1460, 1:100, R&D), anti-TLR4 (Cat# 14-9924-82, 1:100, Invitrogen), anti-GFAP (ab4648, 1:200, abcam), anti-Neu (ab104224, 1:200, abcam), anti-MPO (sc-390109, 1:50, Santa Cruze), anti-CD31 (ab28364, 1:50, abcam), anti-ZO-1 (ab221547, 1:100, abcam) and anti-claudin5 (ab15106, 1:100, abcam) at 4 °C overnight. The next day, the sections were washed with PBS and incubated with the corresponding secondary antibodies: goat anti-rabbit IgG-H&L (Alexa Fluor® 488) (ab150077, 1:1000, abcam), goat anti-rabbit IgG-H&L (Alexa Fluor® 647) (ab150079, 1:1000, abcam), donkey anti-mouse IgG–H&L (Alexa Fluor® 647) (ab150107, 1:1000, abcam), donkey anti-goat IgG H&L (Alexa Fluor® 488) (ab150129, 1:1000, abcam) and donkey anti-mouse IgG H&L (Alexa Fluor® 488) (ab150105, 1:1000, abcam) at room temperature for 1 h. Then, nuclear staining was performed with 4',6-diamidino-2-phenylindole (DAPI; Beyotime Biotech) for 5 min. Images of each section were obtained by laser scanning confocal microscopy (Nikon, Tokyo, Japan), and ImageJ software (Bethesda MD, USA) was used for analysis.

For the immunofluorescence analysis, cells were plated on coverslips in a 6-well cell culture plate. After treatment, the cells were fixed with 4% paraformaldehyde for 20 min, permeabilized with 0.1% Triton X-100 in PBS for 15 min, and blocked with 5% BSA for 40 min. The cells were incubated with anti-NF-κB p65 primary antibody (sc-8008, 1:100, Santa Cruze) at 4 °C overnight, followed by incubation with donkey anti-mouse IgG (H&L) (Alexa Fluor® 488) (ab150105, 1:1000, abcam) for 1 h at room temperature. Then, the nuclei were stained with DAPI for 5 min, after which the cells were visualized and analyzed as described above.

Z-stack confocal images from 30-μm brains sections that were immunostained for Iba-1 were collected at 1-μm intervals using a 60 × objective and microglia morphology was quantified. Briefly, images were obtained from three fields from each slice with two slices per mouse. Consecutive Z-stack images were converted to a maximum intensity projection using Fiji (RRID:SCR_002285), and then transformed to binary and skeletonized images by thresholding to include microglial processes. The skeleton was analyzed by software plugins AnalyzeSkeleton (2D/3D).

To quantify CD16-positive, MPO-positive, TLR4-positive, ZO-1-positive and Claudin-5-positive cells in the lesion boundary zone of the cerebral cortex, we selected at least three sections per mouse with similar areas of ipsilateral cortex and three fields per section. Iba1 co-stained with CD31 images were captured using an SP8 confocal microscope (Lecia, Mannheim, Germany) and 3D reconstruction was performed to show the association of microglia with blood vessels. All analyses were performed in a blinded manner.

### RNA extraction and RT-PCR

Samples from the lesion boundary zone of the cerebral cortex at 72 h after TBI and primary microglia at 12 h after treatment were collected for RT-PCR analysis, respectively. Total mRNA was extracted using TRIzol reagent (Invitrogen, Thermo fisher scientific), and cDNA was synthesized from 1 μg RNA using PrimeScript RT kit (Takara Bio, Tokyo, Japan). qRT-PCR analysis was conducted on an Opticon 2 Real-Time PCR Detection System (Bio-Rad) using SYBR®Green PCR Master Mix (Applied Biosystems, Waltham, MA, USA) and corresponding primers. The specific sequence of primers used were as follows: TNF-α: sense primer 5’-GGT CCC AAC AAG GAG GAG AAG TTC-3’, antisense primer 5’-CCG CTT GGT GGT TTG CTA CGA C-3’; IL-1β: sense primer 5’-CGT GGG ATG ATG ACG ACC TGC-3’, antisense primer 5’-GGA GAA TAC CAC TTG TTG GCT TAT-3’; IL-6: sense primer 5’-GAC AGC CAC TGC CTT CCC TAC TT-3’, antisense primer 5’-CAG AAT TGC CAT TGC ACA ACT CT-3’; iNOS: sense primer 5'-CAG CTG GGC TGT ACA AAC CTT-3', antisense primer 5'-CAT TGG AAG TGA AGC GTT TCG-3'; TLR4: sense primer 5'-CCG CTC TGG CAT CAT CTT CA-3', antisense primer 5'-TGG GTT TTA GGC GCA GAG TT-3'. PCR amplification was performed with a program of 95 °C for 5 min, followed by 40 cycles of 95 °C for 1 min, 62 °C for 30 s, and 72 °C for 30 s. The mRNA expression level of the target gene was normalized to that of the housekeeping gene β-actin, and calculated by the 2^−ΔΔCT^ method. Results are expressed as the mean ± SEM of replicate samples from three independent experiments.

### Statistical analysis

All statistical analyses of the data were processed with Prism 7.0 software (GraphPad, San Diego, CA, USA) in a blinded manner. Data from individual groups were expressed as mean ± SD for in vivo experiments, and as mean ± SEM for in vitro experiments. All the data were characterized by a one-way ANOVA for multiple comparisons or Student’s *t* test (and nonparametric tests). Statistical significance was considered at *p* < 0.05 level.

## Results

### HCQ treatment ameliorated short-term lesion volume and neurologic deficits after TBI

TBI could result in necrosis and the loss of brain tissue. At 3 day post-TBI, we evaluated the effect of HCQ on brain lesion volume. TTC staining showed that the lesions in the cerebral cortex were significantly reduced in the HCQ group compared with the vehicle group (*p* < 0.05, Fig. 1b, c). To determine whether HCQ treatment influenced the recovery of neurological functions in mice after TBI, the mNSS assessment, rotarod test, and grip strength test were performed. The results showed that mice in the TBI group exhibited marked defects in neurological function compared with those of mice in the sham group on days 1 and 3 post-TBI, as indicated by higher mNSS scores (*p* < 0.001, Fig. 1d) and decreased latency times (*p* < 0.001, Fig. 1e) and grip strength (*p* < 0.01, Fig. 1f), which demonstrated that the TBI model was successfully established. However, the mNSS in the HCQ-treated group was similar to that of the vehicle-treated group (*p* > 0.05, Fig. 1d). Furthermore, in the rotarod test, mice in the HCQ-treated group exhibited significant improvements in locomotor function, as demonstrated by increased latency to fall off the rotarod (*p* < 0.01, *p* < 0.05, Fig. 1e) and increased grip strength (*p* < 0.01, Fig. 1f), compared with those in the vehicle-treated group on days 1 and 3 post-TBI. Taken together, these behavioral test data suggest that post-TBI treatment with HCQ facilitates locomotor functional recovery after TBI.

### HCQ decreased the expression of proinflammatory cytokines and attenuated TLR4 and NF-κB p65 activation

**Fig. 2:**
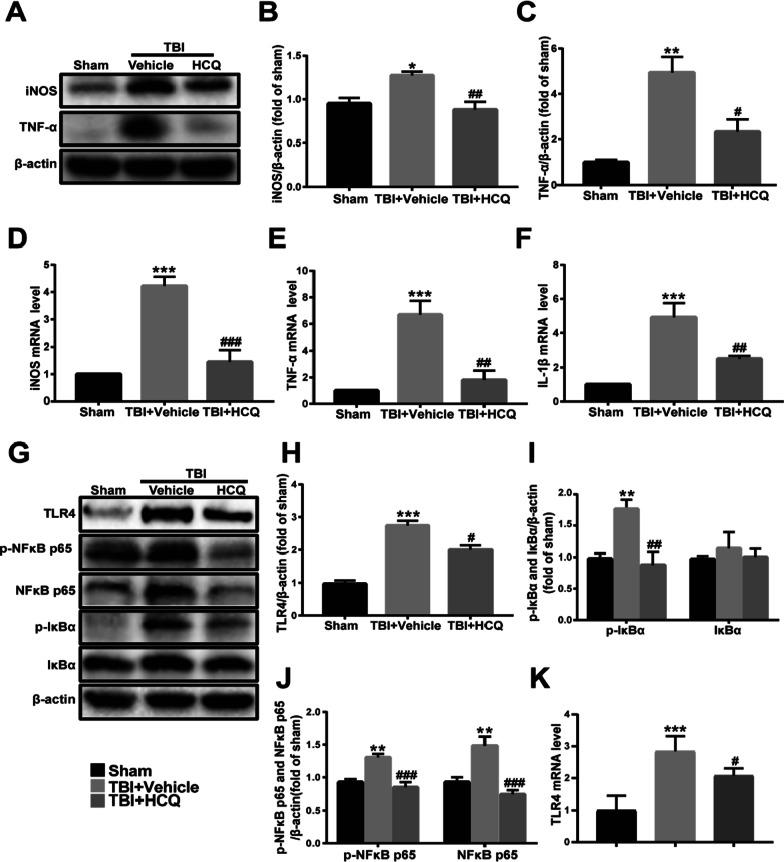
HCQ administration suppressed proinflammatory cytokine expression and TLR4/NF-κB activation in lesioned cortices induced by TBI at 3 day post-TBI. **a** Representative western blot bands of iNOS and TNF-α. **b**, **c** Densitometric quantification of iNOS and TNF-α. *n* = 4 per group. Data are presented as the mean ± SD. *p < 0.05, **p < 0.01 vs. sham group, ^#^*p* < 0.05, ^##^*p* < 0.01 vs. TBI + Vehicle group. **d**–**f** mRNA expression levels of iNOS, TNF-α and IL-1β. n = 4–5 per group. The data are presented as the mean ± SD. ***p < 0.001 vs. sham group, ^**###**^*p* < 0.001, ^**##**^*p* < 0.01 vs. TBI + Vehicle group. **g** Representative western blot bands of TLR4, p-NF-κB p65, NF-κB p65, p-IκBα, and IκBα. **h**–**j** Densitometric quantification of TLR4, p-IκBα, IκBα, p-NF-κB p65, and NF-κB p65, respectively. *n* = 4 per group. The data are presented as the mean ± SD. **p < 0.01, ***p < 0.001 vs. sham group, ^#^*p* < 0.05, ^##^*p* < 0.01, ^**###**^*p* < 0.001 vs. TBI + Vehicle group. **k** mRNA expression levels of TLR4. n = 4 per group. The data are presented as the mean ± SD. ^***^p < 0.001 vs. sham group, ^#^*p* < 0.05 vs. TBI + Vehicle group

Inflammatory cytokine expression plays a crucial role in the inflammatory cascade reaction and neuronal damage after TBI. To determine the effect of HCQ on neuroinflammation following TBI, proinflammatory cytokines, including TNF-α, IL-1β, and iNOS, were evaluated by western blotting or qRT-PCR at 3 day post-TBI. The protein levels of the proinflammatory cytokines iNOS and TNF-α were significantly increased after TBI (*p* < 0.05, Fig. 2a, b and  *p*< 0.01, Fig. 2a, c), while HCQ administration significantly reduced the levels of iNOS (*p* < 0.01, Fig. 2a, b) and TNF-α (*p* < 0.05, Fig. 2a, c). qRT-PCR also revealed similar results: TBI induced high mRNA expression of the proinflammatory cytokines iNOS (*p* < 0.001, Fig. 2d), TNF-α (*p* < 0.001, Fig. 2e) and IL-1β (*p* < 0.001, Fig. 2f), and this effect was markedly inhibited by HCQ (*p* < 0.001, Fig. 2d; *p* < 0.01, Fig. 2e; *p* < 0.01, Fig. 2f). These results demonstrate that HCQ ameliorates the TBI-induced inflammatory response during the acute stage.

TBI may induce activation of the TLR4/NF-κB signaling pathway and inflammatory cytokine expression [39]. To explore whether HCQ inhibits inflammation activation by regulating the TLR4/NF-κB signaling pathway, western blot analysis was performed. The results indicated that the protein levels of TLR4 were significantly increased in the cortex in the TBI + vehicle group compared with the sham group (*p* < 0.001, Fig. 2g, h), and HCQ significantly decreased the TBI-induced protein levels of TLR4 (*p* < 0.05, Fig. 2g, h). We also examined the phosphorylation of NF-κB p65 and IκBα, which are indicators of NF-κB pathway activation. The protein levels of p-IκBα, p-NF-κB p65, and NF-κB p65 were obviously increased in the TBI + vehicle group compared with the sham group (*p* < 0.01, Fig. 2g, i, j), whereas the protein levels of p-IκBα, p-NF-κB p65, and NF-κB p65 in the TBI + HCQ group were significantly lower than those of the TBI + vehicle group (*p* < 0.01, Fig. 2g, i;* p* < 0.001, Fig. 2g, j), and neither TBI nor HCQ treatment significantly affected the protein level of IκBα (*p* > 0.05, Fig. 2g, i), TLR4 mRNA expression level was also elevated in the TBI + Vehicle group (*p* < 0.001, Fig. 2k), and HCQ decreased it (*p* < 0.05, Fig. 2k). Taken together, these results indicate that after TBI, HCQ administration inhibits the inflammatory response, affects the expression of TLR4 and inhibits the activation of TLR4/NF-κB signaling pathway.

### HCQ suppressed microglial activation and immune cell infiltration

**Fig. 3:**
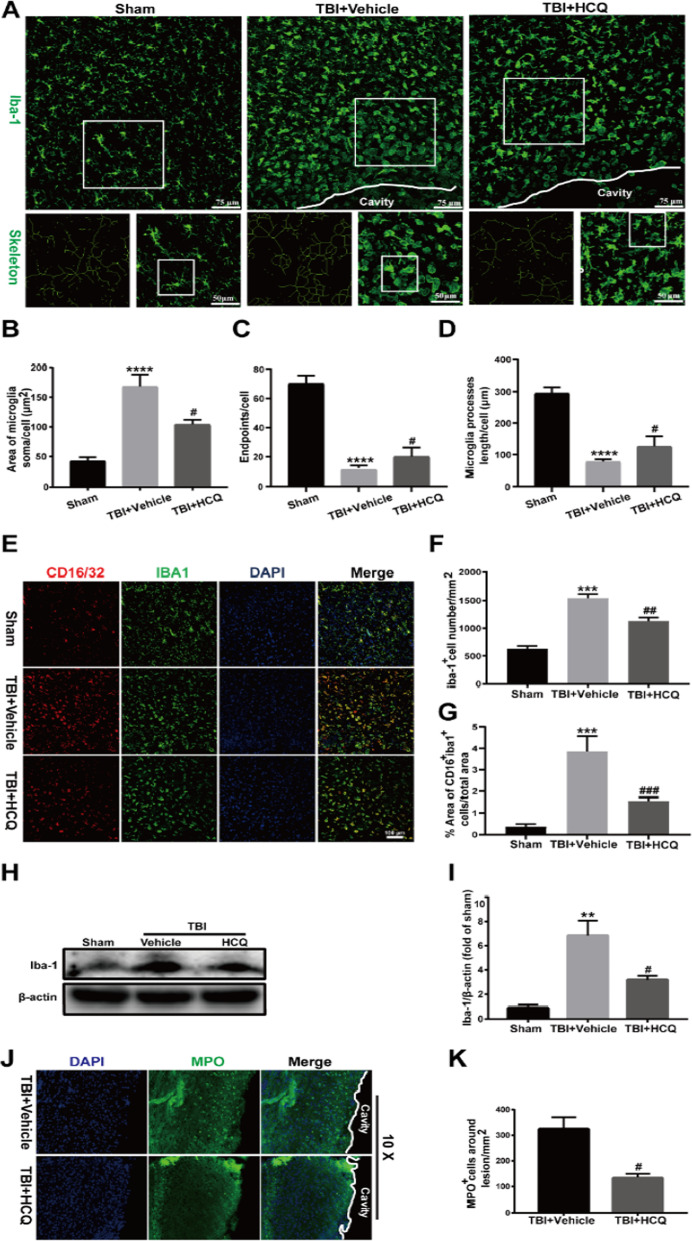
HCQ administration alleviated microglial activation and neutrophil infiltration in the ipsilateral cortex at 3 day post-TBI. **a** Representative images of microglial activation, as shown by Iba-1 staining in lesioned cortices. **b**–**d** Quantification of the microglial soma areas, branch endpoints and microglial process lengths. *n* = 5 per group. The data are presented as the mean ± SD. ****p < 0.0001 vs. sham group, ^#^*p* < 0.05 vs. TBI + Vehicle group. **e**–**g** Immunofluorescent staining and statistical analysis of CD16/32 and Iba1. *n* = 5 per group. The data are presented as the mean ± SD. ***p < 0.001 vs. sham group, ^##^*p* < 0.01, ^**###**^*p* < 0.001 vs. TBI + Vehicle group. **h**, **i** Representative western blot bands and densitometric quantification of Iba-1 expression. *n* = 4 per group. The data are presented as the mean ± SD. **p < 0.01 vs. sham group, ^#^*p* < 0.05 vs. TBI + Vehicle group. **j** Representative images of MPO staining in the ipsilateral cortex. **k** Quantification of MPO-positive cells. *n* = 5 per group. The data are presented as the mean ± SD. ^#^*p* < 0.05 vs. TBI + Vehicle group

Microglial activation plays an important role in neuroinflammation after TBI. The microglial shift from a ramified state to an activated state is marked by reduced process length, branch endpoint and swollen soma [33]. To investigate the potential role of HCQ in microglial activation, immunofluorescence staining of the microglial marker Iba1 was performed. Assessment of microglia skeleton showed that microglia in the TBI + Vehicle group presented larger soma, decreased branch endpoints and shorter process lengths (*p* < 0.0001, Fig. 3a–d). However, HCQ alleviated TBI-induced morphological changes in microglia (*p* < 0.05, Fig. 3a–d). CD16 was used to label pro-inflammatory microglia, M1 microglia. Double immunofluorescent staining of CD16 with a microglia/macrophage marker Iba1 confirmed a decreased M1 polarization 3 days after TBI in HCQ-treated mice as compared to vehicle treated mice (*p* < 0.001, Fig. 3e, g). HCQ also reduced the Iba1-positive cell density (*p* < 0.01, Fig. 3e, f). These indicated that TBI-induced microglial activation was alleviated by HCQ. In addition, western blotting was used to assess the protein expression of Iba1. Consistently, TBI induced high expression of Iba1 (*p* < 0.01, Fig. 3h, i), and HCQ treatment attenuated these levels (*p* < 0.05, Fig. 3h, i). At 3 day post-TBI, we examined neutrophil infiltration by MPO staining. HCQ administration reduced the number of MPO-positive cells in the ipsilateral cortex after TBI (*p* < 0.05, Fig. 3j, k).

**Fig. 4:**
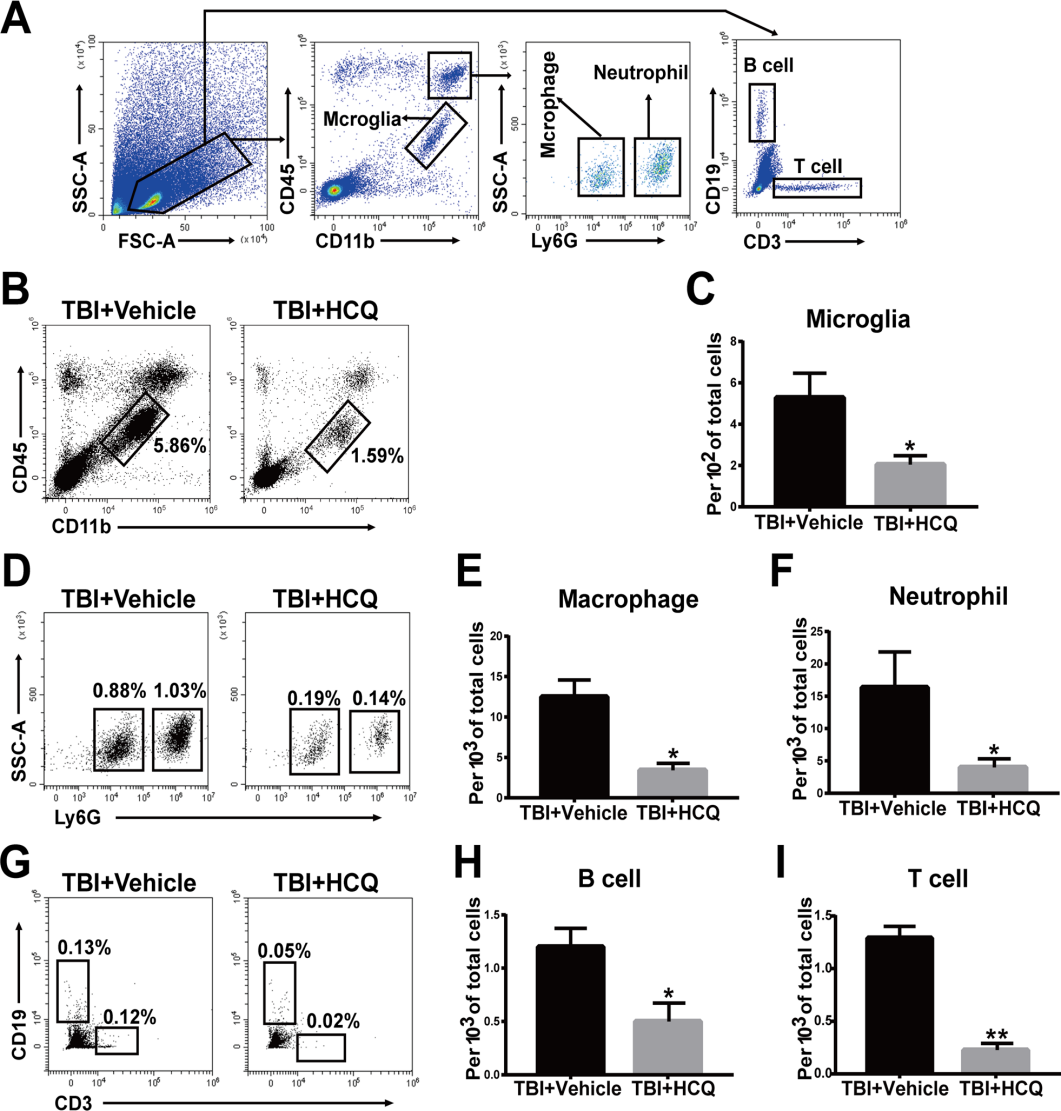
Effect of HCQ on resident microglia and immune cell infiltration in the brain after TBI.** a** The gating strategy of brain-resident microglia (CD11b^+^CD45^int^) and infiltrating immune cells, including macrophages (CD11b^+^CD45^hi^Ly6G^−^), neutrophils (CD11b^+^CD45^hi^Ly6G^+^), B cells (CD45^hi^CD3^−^CD19^+^), and T cells (CD45^hi^CD3^+^CD19^−^). **b**, **c** Representative FACS plots and quantification of the percentage of resident microglia among brain cells. n = 4 for TBI + Vehicle group and n = 5 for TBI + HCQ group. The data are presented as the mean ± SD. ^*^p < 0.05 vs. TBI + Vehicle group. **d** Representative FACS plots showing macrophages and neutrophils. **e**, **f** Quantification of the percentage of macrophages and neutrophils among brain cells. n = 4 for TBI + Vehicle group and n = 5 for TBI + HCQ group. ^*^p < 0.05 vs. TBI + Vehicle group. **g** Representative FACS blots showing B cells and T cells. **h, i** Quantification of the percentage of B cells and T cells among brain cells. *n* = 4 for TBI + Vehicle group and *n* = 5 for TBI + HCQ group. The data are presented as the mean ± SD. *p < 0.05, **p < 0.01vs. TBI + Vehicle group

In addition, flow cytometry was performed to assess the accumulation of microglia, which were defined as CD11b^+^ CD45^int^, and infiltrating immune cells, such as macrophages (CD11b^+^ CD45^hi^ Ly6G^−^), neutrophils (CD11b^+^ CD45^hi^ Ly6G^+^), B cells (CD45^hi^ CD3^−^ CD19^+^), and T cells (CD45^hi^ CD3^+^ CD19^−^), in the brain at 3 day post-TBI (Fig. 4a). Flow cytometric analysis revealed that HCQ administration dramatically reduced the numbers of microglia (*p* < 0.05, Fig. 4b, c) and infiltrated peripheral immune cells, such as macrophages (*p* < 0.05, Fig. 4d, e), neutrophils (*p* < 0.05, Fig. 4d, f), B cells (*p* < 0.05, Fig. 4g, h), and T cells (*p* < 0.01, Fig. 4g, i), compared to vehicle treatment. Collectively, these findings suggest that after TBI, HCQ treatment alleviated microglial activation and peripheral immune cell infiltration.

### HCQ administration did not alter immune cell numbers in the spleen or blood

**Fig. 5:**
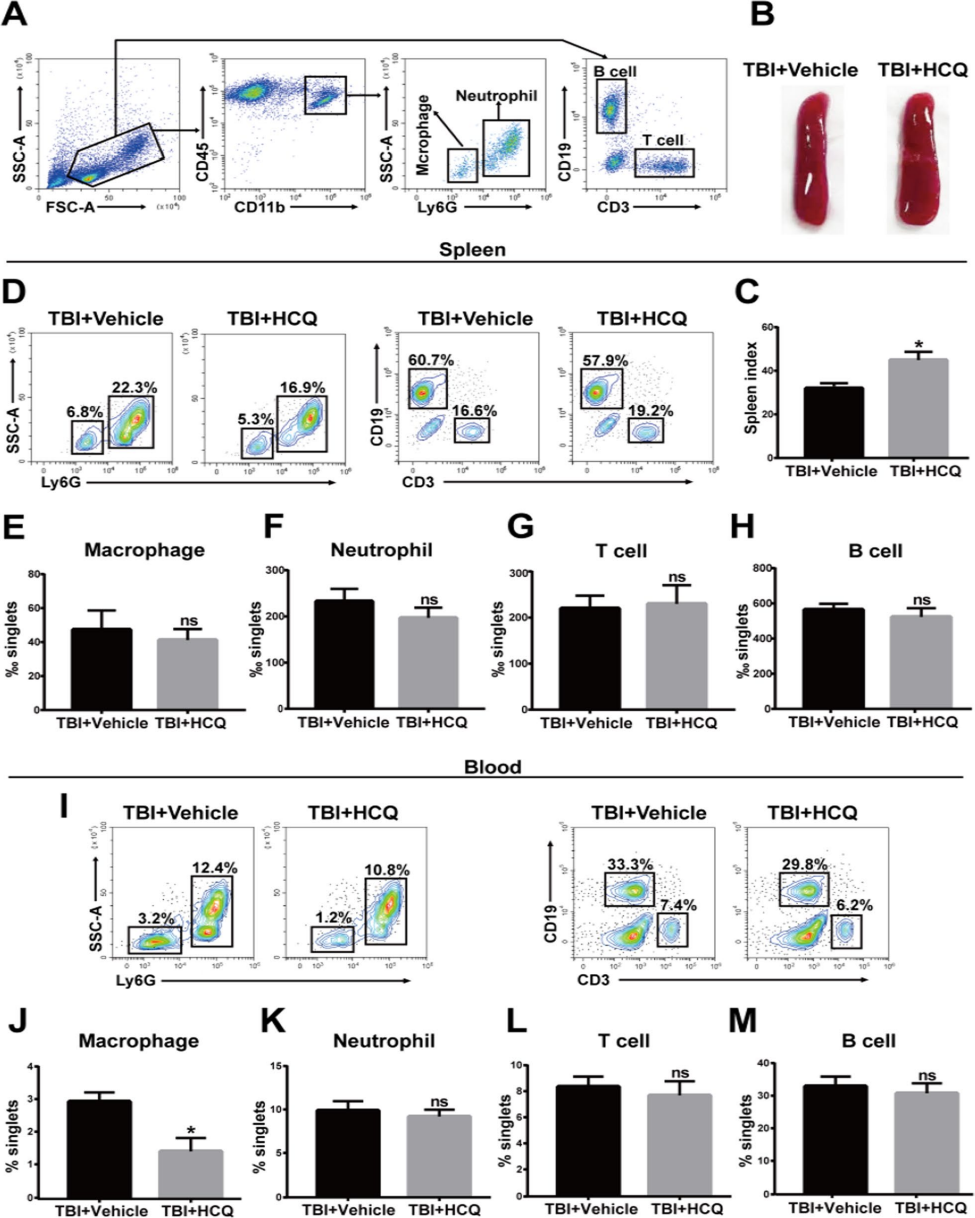
HCQ administration did not alter immune cell numbers in the spleen or blood. **a** The gating strategy of immune cells, including macrophages (CD11b^+^CD45^hi^Ly6G^−^), neutrophils (CD11b^+^CD45^hi^Ly6G^+^), B cells (CD45^hi^CD3^−^CD19^+^), and T cells (CD45^hi^CD3^+^CD19^−^), in the spleen and blood. **b, c** Representative images of the spleen and spleen indices of vehicle- and HCQ-treated TBI mice. n = 4 per group. The data are presented as the mean ± SD. *p < 0.05 vs. TBI + Vehicle group. **d** Representative FACS plots showing macrophages, neutrophils, T cells, and B cells in the spleen. **e**–**h** Quantification of the percentage of macrophages, neutrophils, T cells, and B cells in the spleen. *n* = 4 per group. The data are presented as the mean ± SD. ns: no significant difference.** i** Representative FACS panels of immune cells macrophage, neutrophils, T cells, and B cells in blood. **j**–**m** Quantification of the percentage of immune cells macrophage, neutrophils, T cells, and B cells in blood. *n* = 4 per group. The data are presented as the mean ± SD. *p < 0.05 vs. TBI + Vehicle group, ns: no significant difference

Disruption of the BBB facilitates the recruitment of immune cells from the spleen and blood to the site of injury in the brain and induces subsequent repair processes [40]. Therefore, the immune cell composition in the blood and spleen was evaluated by flow cytometry at 3 day post-TBI. Macrophages (CD11b^+^CD45^hi^Ly6G^−^), neutrophils (CD11b^+^CD45^hi^Ly6G^+^), B cells (CD45^hi^CD3^−^CD19^+^), and T cells (CD45^hi^CD3^+^CD19^−^) were identified with specific markers (Fig. 5a). In addition, the mice were sacrificed, and the spleen mass was measured and normalized to body weight and is expressed as the spleen index (spleen weight (mg) × 10/body weight (g)). Interestingly, HCQ treatment slightly increased the spleen index compared to that in the vehicle-treated TBI group (*p* < 0.05, Fig. 5b, c). Unexpectedly, there was no significant difference observed in the macrophage, neutrophil, B cell, and T cell counts in the spleen (*p* > 0.05, Fig. 5d–h) between vehicle- and HCQ-treated animals. In addition, the protein expression levels of TLR4, p-NF-κB p65, and NF-κB p65 in the spleen were detected by western blot. We showed that TLR4 (*p* < 0.05, Additional file 1: Fig. S1a, b), p-NF-κB p65 (*p* < 0.01, Additional file 1: Fig. S1a, c), and NF-κB p65 (*p* < 0.01, Additional file 1: Fig. S1a, d) expression were elevated by TBI. HCQ significantly reduced TBI-induced protein levels of p-NF-κB p65 (*p* < 0.01, Additional file 1: Fig. S1a, c) and NF-κB p65 (*p* < 0.01, Additional file 1: Fig. S1a, d), but the amount of TLR4 was not statistically decreased (*p* > 0.05, Additional file 1: Fig. S1a, b). In the blood, HCQ decreased the number of macrophages (*p* < 0.05, Fig. 5i, j) but had no effect on other peripheral immune cell numbers (*p* > 0.05, Fig. 5i, k–m).

### HCQ attenuated TLR4 expression and NF-κB nuclear translocation and reduced LPS-induced proinflammatory cytokine expression in primary microglial cells

**Fig. 6:**
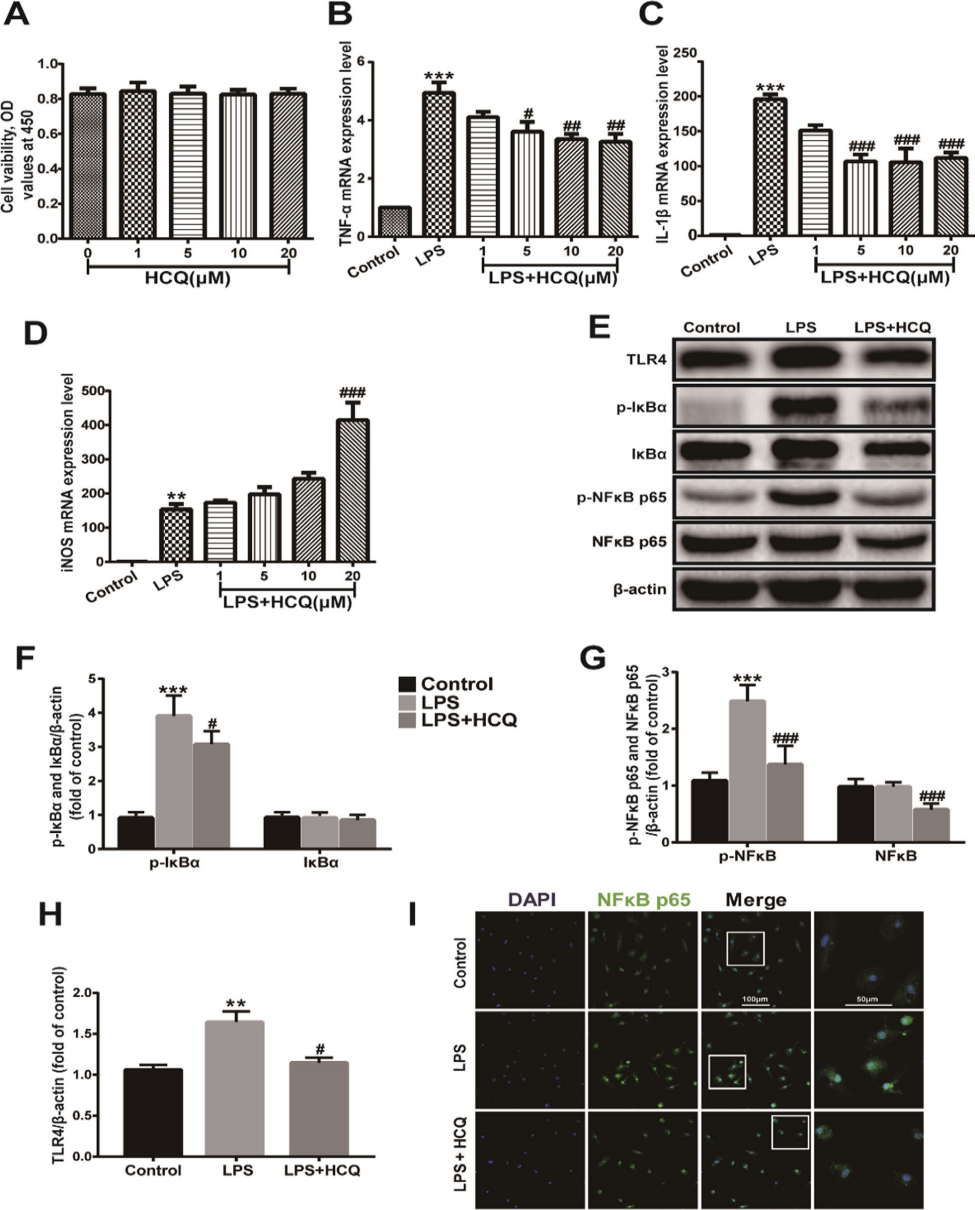
HCQ reduced LPS-induced pro-inflammatory cytokines expression and attenuated TLR4/NF-κB activation in primary microglial**. a** Cell vaibility of primary microglial treated with different concentration of HCQ. **b**–**d** mRNA expression level of TNF-α, IL-1β and iNOS in LPS-stimulated primary microglial. *n* = 4 per group. The data are presented as the mean ± SEM. ***p < 0.001 vs. Control group, ^#^*p* < 0.05, ^##^*p* < 0.01, ^**###**^*p* < 0.001 vs. LPS group. **e** Representative western blot bands of TLR4, p-IκBα, IκBα, p-NF-κB and NF-κB. **f**–**h** Densitometric quantification of TLR4, p-IκBα, IκBα, p-NF-κB and NF-κB. *n* = 4 per group. The data are presented as the mean ± SEM. **p < 0.01, ***p < 0.001 vs. Control group. ^#^*p* < 0.05, ^###^*p* < 0.001 vs. LPS group. **i** Representative photographs of immunofluorescence staining of NF-κB. HCQ inhibited NF-κB translocation to the nucleus in LPS-stimulated primary microglial

In vivo, HCQ reduced TBI-induced TLR4 expression levels. Immunofluorescent co-staining of TLR4 with Iba1 (microglia/macrophage marker), GFAP (astrocyte marker), Neu (neuron marker) showed that TLR4 expression was mainly observed in microglia/macrophage cells, and a small number of astrocytes and neurons also expressed TLR4 (*p* < 0.01, Additional file 1: Fig. S2a, c) at 3 day post-TBI. It suggested that the effect of HCQ on TLR4 expression was concentrated in microglia, as evidenced by immunofluorescence staining (*p* < 0.01, Additional file 1: Fig. S2b, d, e). To further evaluate the effects of HCQ on proinflammatory cytokine expression and the regulation of the TLR4/NF-κB signaling pathway, primary microglial cells were exposed to LPS. Cell viability assays were first used to assess whether HCQ was toxic toward primary microglia. As expected, HCQ did not induce primary microglial toxicity at concentrations up to 20 μM (*p* > 0.05, Fig. 6a). Then, we used RT-PCR to investigate the potential regulatory effects of HCQ on proinflammatory responses. In vitro, LPS induced high mRNA expression levels of TNF-α (*p* < 0.001, Fig. 6b), IL-1β (*p* < 0.001, Fig. 6c) and iNOS (*p* < 0.01, Fig. 6d). HCQ significantly reduced the LPS-induced mRNA levels of the proinflammatory cytokines TNF-α (*p* < 0.05 or 0.01, Fig. 6b) that was consisted with the previous study [28], and HCQ reduced IL-1β (all *p* < 0.001, Fig. 6c) in a dose-dependent manner. However, HCQ did not reduce the high expression of iNOS, and 20 μM HCQ markedly increased iNOS expression (*p* < 0.001, Fig. 6d). Consistent with the in vivo results, LPS significantly increased p-IκBα (*p* < 0.001, Fig. 6e, f), p-NF-κB p65 (*p* < 0.001, Fig. 6e, g), and TLR4 (*p* < 0.01, Fig. 6e, h) protein levels compared with those of the control group. HCQ significantly downregulated LPS-induced p-IκBα (*p* < 0.05, Fig. 6e, f), p-NF-κB p65 (*p* < 0.001, Fig. 6e, g), and TLR4 (*p* < 0.05, Fig. 6e, h) levels in primary microglial cells. Interestingly, the level of IκBα was not affected (*p* > 0.05, Fig. 6e, f), while the protein level of NF-κB p65 was decreased by HCQ treatment (*p* < 0.001, Fig. 6e, g). Moreover, the immunofluorescence staining results demonstrated that NF-κB p65 protein expression levels in the nucleus increased after LPS stimulation and that HCQ inhibited the translocation of NF-κB p65 into the nucleus (Fig. 6i). These data suggest that HCQ may regulate activation of the TLR4/NF-κB pathway to modify LPS-induced proinflammatory responses.

### HCQ administration attenuated BBB disruption after TBI

**Fig. 7:**
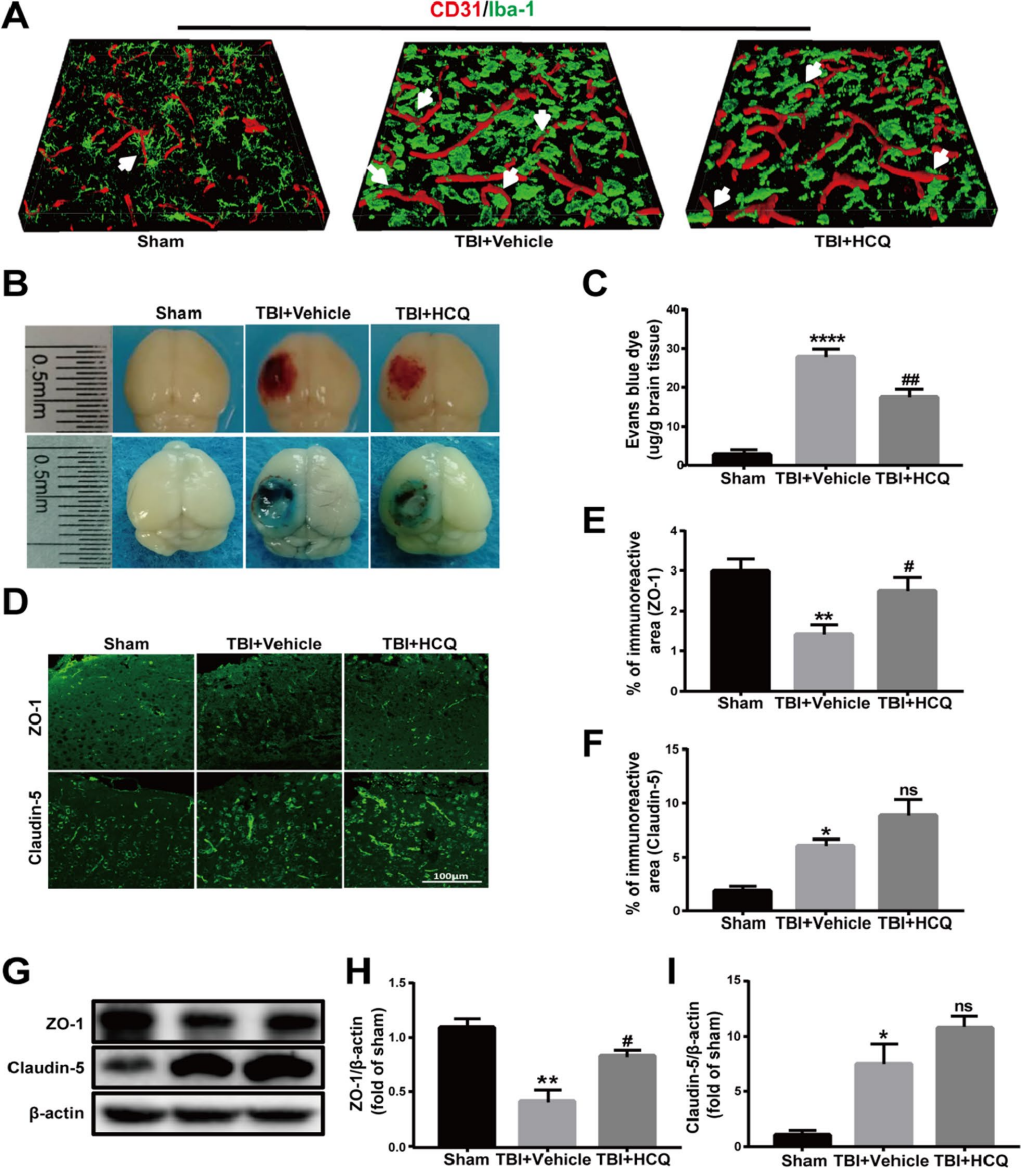
HCQ administration attenuated BBB disruption at 3 day post-TBI. **a** Representative 3D reconstructed images of the colocalization of CD31 (red) and Iba1 (green). **b** Representative images of brain tissue and EB leakage at 3 day post-TBI. **c** Quantification of EB extravasation. n = 5 per group. Data are presented as the mean ± SD. ****p < 0.0001 vs. sham group, ^##^*p* < 0.01, vs. TBI + Vehicle group. **d**–**f** Representative photographs showing immunofluorescence staining and the quantitative analysis of ZO-1 and claudin-5 expression in the lesioned boundary at 3 day postinjury. n = 5 per group. The data are presented as the mean ± SD. *p < 0.05, **p < 0.01 vs. sham group, ^#^*p* < 0.05 vs. TBI + Vehicle group. ns: no significant difference. **g**–**i** Representative western blot bands and densitometric quantification of ZO-1 and claudin-5 expression. *n* = 4 per group. The data are presented as the mean ± SD. *p < 0.05, **p < 0.01 vs. sham group, ^#^*p* < 0.05 vs. TBI + Vehicle group. ns: no significant difference

Previous studies have shown that posttraumatic inflammatory cascades and overactivated perivascular microglia contribute to the disintegration and degradation of blood vessels and eventually lead to BBB breakdown after brain injury [32, 41]. We further investigated the relationship between microglia and vessels after TBI by double-staining of Iba1 and CD31. As Fig. 7a shows, in the sham group, ramified microglia were evenly distributed and intertwined with the blood vessels. However, after TBI, microglia were activated and developed an ameboid morphology with an enlarged cell soma and retracted, thick processes that were in direct contact with blood vessels. Activated microglia could clear blood vessel fragments but also engulf intact blood vessels, and release inflammatory factors that cause vessel damage.

Since our results showed that HCQ inhibited neuroinflammation and microglial activation, we, therefore, explored whether HCQ had a positive effect on BBB disruption. BBB integrity was evaluated by EB dye extravasation and errhysis. At 3 day post-TBI, we observed that errhysis and EB dye extravasation in the ipsilateral hemisphere in the TBI group were significantly increased compared with those in the sham group (*p* < 0.0001, Fig. 7b, c), indicating severe breakdown of the BBB. However, these increases in EB levels and errhysis were markedly attenuated by HCQ treatment (*p* < 0.01, Fig. 7b, c). Tight junction proteins are essential in maintaining the BBB. Therefore, the expression of the tight junction proteins ZO-1 and claudin-5 was quantified using immunofluorescence staining and western blotting at 3 day postinjury. The immunostaining results showed that the expression of ZO-1 in the TBI group was decreased compared with that in the sham group (*p* < 0.01, Fig. 7d, e), and this decrease was rescued by HCQ treatment (*p* < 0.05, Fig. 7d, e). The expression of claudin-5 in the TBI group was higher than that in the sham group (*p* < 0.05, Fig. 7d, f), which was consistent with a previous report [42] and was probably due to the stress response. HCQ treatment further increased the expression of claudin-5 compared with that in the TBI group, but the difference was not significant (*p* > 0.05, Fig. 7d, f). Similar results were observed by western blotting (*p* < 0.01 or 0.05, Fig. 7g, h; and *p* < 0.05 or *p* > 0.05, Fig. 7i). Overall, these results indicate that TBI-induced BBB disruption can be rescued by HCQ administration. The molecular mechanism of HCQ suppressing MMPs expression may involve in the function of HCQ protecting BBB after TBI.

### Combined administration of HCQ and TAK-242 did not enhance the effects of HCQ in the expression of TLR4, p-NF-κB p65, NF-κB p65, pro-inflammatory cytokines and neurological function

**Fig. 8:**
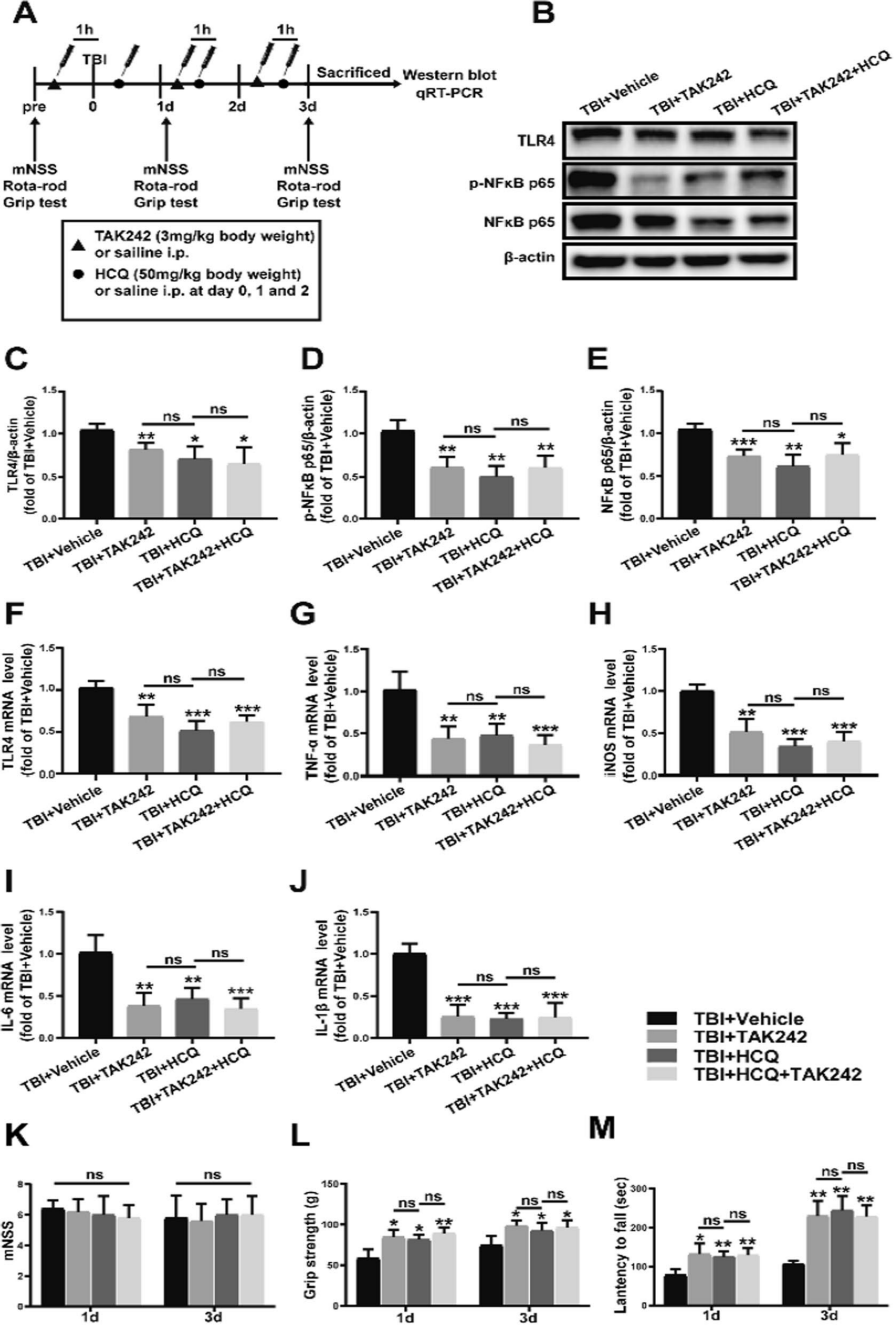
Combined administration of HCQ and TAK-242 did not enhance the effects of HCQ in the expression of TLR4, p-NF-κB p65, NF-κB p65, pro-inflammatory cytokines and neurological function. **a** Schematic diagram of the experimental design. **b**–**e** Representative western blot bands and densitometric quantification of TLR4, p-NF-κB p65 and NF-κB p65. *n* = 5 per group. The data are presented as the mean ± SD. ^*^p < 0.05, ^**^p < 0.01 and ^***^p < 0.001 vs. TBI + Vehicle group. ns: no significant difference. **f**–**j** mRNA expression levels of TLR4, TNF-α, iNOS, IL-6 and IL-1β. n = 6 per group. The data are presented as the mean ± SD. **p < 0.01, ***p < 0.001 vs. TBI + Vehicle group. ns: no significant difference. **k**–**m** Neurological recovery was evaluated by mNSS, the rotarod test, and the grip strength at 1 and 3 day post-TBI. *n* = 11 per group. The data are presented as the mean ± SD. *p < 0.05, **p < 0.01 vs. TBI + Vehicle group

To further determine whether TLR4/NF-κB signaling was required for HCQ-mediated anti-inflammatory and improved neural pathology effects, we assessed the effect of TAK-242, a TLR4 inhibitor, on both TBI and HCQ-treated mice. The protein levels of TLR4, p-NF-κB p65 and NF-κB p65 were decreased in the TBI + TAK242 (*p* < 0.01, *p* < 0.01 and *p* < 0.001, Fig. 8b–e), TBI + HCQ (*p* < 0.05, *p* < 0.01 and *p* < 0.01, Fig. 8b–e) and TBI + TAK-242 + HCQ group (*p* < 0.05, *p* < 0.01 and *p* < 0.05, Fig. 8b–e) as compared with the TBI + Vehicle group. There was no significant difference between TBI + HCQ and TBI + TAK-242 + HCQ in proteins level of TLR4, p-NF-κB p65 and NF-κB p65 (*p* > 0.05, Fig. 8b–e). The mRNA levels of TLR4 and pro-inflammatory cytokines TNF-α, iNOS, IL-6 and IL-1β were reduced in TBI + TAK242 (*p* < 0.01, *p* < 0.01, *p* < 0.01, *p* < 0.01 and *p* < 0.001, Fig. 8f–j), TBI + HCQ (*p* < 0.001, *p* < 0.01, *p* < 0.001, *p* < 0.01 and *p* < 0.001, Fig. 8f–j) and TBI + TAK-242 + HCQ group (*p* < 0.001, Fig. 8f–j) as compared with the TBI + Vehicle group. No significant difference was observed in these mRNA levels between TBI + HCQ and TBI + TAK-242 + HCQ group (*p* > 0.05, Fig. 8f–j). Combined administration of TAK-242 and HCQ had no effect on mNSS (*p* > 0.05, Fig. 8k), but increased latency to fall off the rotarod (*p* < 0.01, *p* < 0.05 Fig. 8l) and grip strength (*p* < 0.01, Fig. 8m), which was similar with HCQ treatment (*p* > 0.05, Fig. 8k–m). These results suggested that attenuating the TLR4/NF-κB signaling pathway is beneficial in TBI recovery, and the inhibition of TLR4 signaling has a similar effect as HCQ treatment after TBI, indicating that the effect of HCQ on TBI maybe through the TLR4/NF-κB signaling pathway.

### The neuroprotective effect of HCQ was extended to 6 h post-TBI

**Fig. 9:**
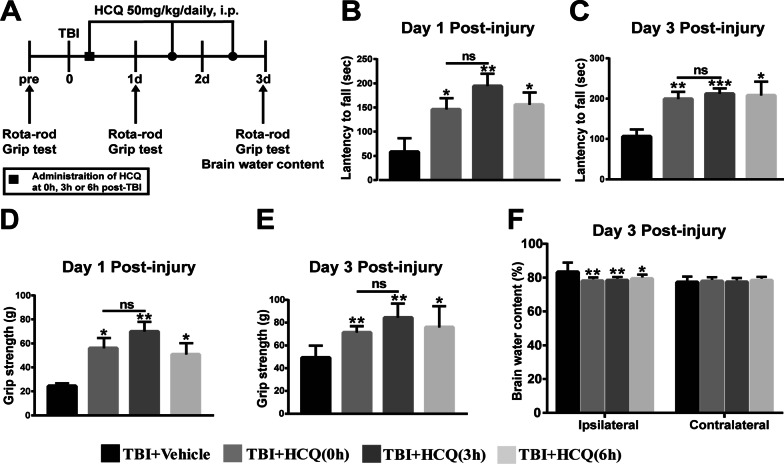
Efficacy of HCQ was extended to 6 h post-TBI.** a** Schematic diagram of the experimental design. HCQ was administered immediately or at 3 h or 6 h after TBI. Neurological function was evaluated by the rotarod test and grip strength test at 1 and 3 day post-TBI. Brain water content was analyzed 3 days after TBI. **b**, **c** Rotarod test at 1 and 3 day post-TBI. n = 5 per group. The data are presented as the mean ± SD. *p < 0.05, **p < 0.01, ***p < 0.001 vs. TBI + Vehicle group. ns: no significant difference. **d**, **e** Grip strength test at 1 and 3 day post-TBI. *n* = 5 per group. The data are presented as the mean ± SD. *p < 0.05, **p < 0.01 vs. TBI + Vehicle group. ns: no significant difference. **f** Brain water content at 3 day post-TBI. *n* = 5 per group. The data are presented as the mean ± SD. *p < 0.05, **p < 0.01 vs. TBI + Vehicle group

To investigate the therapeutic window of HCQ treatment after TBI, HCQ was administered to the mice 0 h, 3 h, and 6 h after TBI, and then, the rotarod and grip strength tests were performed (Fig. 9a). As shown in Fig. 9b–e, delayed administration of HCQ at 3 h and 6 h after TBI significantly attenuated neurological deficits on day 1 (*p* < 0.01 or 0.05, Fig. 9b and *p* < 0.01 or 0.05, Fig. 9d) and day 3 (*p* < 0.001 or 0.05, Fig. 9c and *p* < 0.01 or 0.05, Fig. 9e) post-TBI and mitigated brain edema at day 3 compared with that in the TBI group (*p* < 0.01 or 0.05, Fig. 9f). In contrast to the results observed when HCQ was administered immediately after TBI, HCQ treatment at 3 h post-TBI seemed to have a better therapeutic effect, but there were no observable differences (all *p* > 0.05, Fig. 9b–e). These results suggest that the therapeutic window for HCQ to protect against TBI can be extended to 6 h post-TBI.

## Discussion

HCQ was FDA-approved for lupus in 1955, and it is an “old” drug that has already been used clinically for decades [21] due to its anti-inflammatory and immunomodulatory properties [25, 26]. The safety of HCQ has been universally verified in clinical practice [27]. However, the effects of HCQ on inflammation and the infiltration of immune cells and the potential mechanism after TBI remain unclear. The present study demonstrates that HCQ administration significantly ameliorates TBI-induced brain neurological deficits. The novel findings were that (1) HCQ alleviated neuroinflammation, the accumulation of microglia and immune cell infiltration in the brain but did not significantly alter immune cell numbers in the spleen or blood. (2) HCQ attenuated BBB disruption and brain edema and upregulated tight junction protein expression. (3) HCQ reduced proinflammatory cytokine expression, and the underlying mechanism, at least in part, involved suppressing the TLR4/NF-κB signaling pathway.

Microglia are known as resident innate immune cells in the CNS and are considered pivotal mediators of neuroinflammatory processes [20]. After TBI, overactivated microglia produce various inflammatory cytokines (IL-1β, IL-6, and TNF-α) and inflammatory mediators (COX-2 and iNOS) [43]. IL-1 production plays a negative role in clinical outcomes following TBI. The levels of IL-1β are one of the most frequently evaluated inflammatory factors in TBI research and have been shown to increase following brain injury [14]. In our current study, the in vivo and in vitro findings demonstrated that HCQ markedly reduced the expression level of IL-1β, which was increased by TBI. TNF acts as a major coordinator of the inflammatory response, and the role of TNF signaling in the pathophysiology of brain injury and disease is controversial. Initial work suggested that TNF exerts adverse effects during the acute phase post-TBI but has a more protective effect in the chronic phase [6, 44]. However, it has been reported that TNF is essential in protecting against early mortality within the first week after injury [45]. We found that HCQ significantly reduced TBI-induced TNF-α upregulation. In addition, HCQ decreased the inflammatory cytokine IL-1β and the inflammatory mediator iNOS. A previous study demonstrated that CQ reduced the expression of inflammatory cytokines, such as TNF-α, IL-6 and IL-1β, and protected against liver damage. Tang et al. [25] found that HCQ inhibited the expression levels of IL-1β, IL-6, and TNF-α to protect against ischemia/reperfusion (I/R)-induced renal injury, which is consistent with our observations.

TBI causes cellular injury and BBB disruption that facilitates the infiltration of peripheral immune cells, including macrophages, neutrophils, T cells and B cells. Post-TBI, brain-resident microglia and peripheral immune cells are immediately activated and infiltrate the site of injury, which exacerbates neuroinflammation through the generation and release of large amounts of inflammatory cytokines [37]. Recently, HCQ has been shown to have T-cell–specific effects. Goldman et al. reported that HCQ could inhibit T-cell antigen receptor (TCR) mediated calcium mobilization and reduced a B-cell antigen receptor calcium signal, indicating a new mechanism for the immunomodulatory properties of HCQ [46]. In our study, we found that HCQ effectively suppressed immune cell infiltration (e.g., macrophages, B cells, and T cells) and restrained brain-resident microglial activation in the injured brain. However, no significant effect on immune cell numbers in the spleen or blood was observed in our study. As we performed no functional experiments on T cells, B cells, and macrophages, we could not conclude that the role in the anti-inflammatory effect of HCQ was solely due to changes in cell numbers.

Neuroinflammation can alter BBB permeability directly via cytokine-mediated activation of metalloproteinases or the disruption of tight junctions [45]. The protection of BBB integrity following TBI presents another potential mechanism of HCQ-induced neuroprotection. Various studies have demonstrated that regulatory molecules can increase tight junction expression and improve BBB integrity. Hemocoagulase agkistrodon (HCA) administration reduced BBB disruption by regulating the expression of tight junction proteins, including ZO-1, occludin and claudin-5, in acute TBI [47]. In the present study, we observed that HCQ administration significantly alleviated TBI-induced degradation of the tight junction protein ZO-1. However, unexpectedly, the tight junction protein claudin-5 was increased in the vehicle-treated TBI group, and HCQ increased its expression, but the difference was not significant. Matrix metalloproteinases (MMPs) are a family of metalloproteinase endonucleases. They can degrade most of the extracellular matrix components [48]. MMPs, including MMP-1, -3, and -9, could cause the destruction of the tight junctions between brain tissue capillaries and damage the BBB in TBI [49]. Previous studies demonstrated that autophagy inhibitor HCQ suppressed the expression of MMP-9 and MMP-2 [50, 51]. In the neurofibromatosis dermal cell lines, MMP-1 protein was downregulated, and this decrease can be restored by the HCQ [52]. The molecular mechanism of HCQ suppressing MMPs expression may involved in the function of HCQ protecting BBB after TBI.

TLR4 is an extensively studied TLR that is linked to various pathophysiological conditions. TLR4 activation is essential for neuroinflammatory responses. Following the initial traumatic insult, damaged cells release of DAMPs, which bind to and activate TLR4, ultimately inducing NF-κB activation and nuclear translocation, which in turn drives the expression of innate immune and inflammatory genes to cause neuroinflammation [53]. Ciprofloxacin and levofloxacin attenuate the microglial inflammatory response via the TLR4/NF-κB pathway [54]. TLR4 may be an important therapeutic target for neuroinflammatory injury post-TBI. It has been reported that in a rat model of TBI, TLR4 knockdown attenuates the neuroinflammatory response and brain injury [55]. VX765, a known caspase-1 inhibitor, inhibited inflammatory activity through the TLR4/NF-κB pathway [56]. In the present study, we observed that HCQ could alleviate neuroinflammation and immune cell infiltration in the brain, thus promoting neurological function recovery and BBB restoration. Consistent with a previous study, these findings indicated that HCQ inhibited the production of inflammatory cytokines in response to TLR4 activation in macrophages, and HCQ treatment decreased TLR4 mRNA and protein expression in BeWo cells [57]. Tang et al. demonstrated that HCQ attenuated renal I/R injury through the inhibition of NF-κB signaling and suppression of cathepsin B- and cathepsin L-mediated NLRP3 inflammasome activation [25]. It has been previously reported that CQ could protect against liver damage in sepsis-induced acute kidney injury by suppressing the expression of the inflammatory cytokines TNF-α and IL-10 through TLR9 inhibition [58]. CQ alleviated NF-κB and MAPK activation and suppressed LPS-induced NLRP3 inflammasome activation in murine bone marrow-derived macrophages (BMDMs), as shown by the reduced expression of IL-1β, IL-18, and Nlrp3 [19].

Our study showed that HCQ improved neurological function and attenuated BBB disruption and brain edema after TBI. HCQ, as a well-known autophagy inhibitor, could deacidify the lysosome and inhibit the autophagosome fusion with lysosomes, thus terminally blocking the autophagic flux [59, 60]. It has been reported as an autophagy inhibitor in various disease, including Alzheimer’s disease [61], brain ischemia [62], as well as TBI [31]. The mechanism underlying the neuroprotective effects of HCQ on TBI, is through the prevention of autophagic neuronal death [31]. In the present study, we did not check the ability of HCQ as an autophagy inhibitor, thus we cannot rule out this function as potentially influencing our observation. Moreover, we demonstrated that HCQ exerts anti-inflammatory effects on mice after TBI, as indicated by HCQ treatment obviously reducing the expression levels of proinflammatory cytokines; the possible mechanisms, at least in part, involve the TLR4/NF-κB signaling pathway and the alleviation of immune cell infiltration. In addition, HCQ markedly attenuated BBB disruption and brain edema by upregulating tight junction protein expression.

There are some limitations in our study. The present study aimed to investigate the effects and potential function of HCQ in neuroinflammation and the BBB during the acute phase of TBI. A longterm study of HCQ after TBI is still needed in the future. Another limitation is that we did not address all pathways and only looked at TLR4 expression in TBI. Several other redundant pathways maybe involved, such as the HMGB1/RAGE/NF-κB axis. Besides, the complement pathway represents a key element in the inflammatory response, and it contributes to neuronal cell death, edema, and infiltration of inflammatory cells after brain injury [63–65]. HCQ has been reported to inhibit complement C5a levels in the antiphospholipid syndrome (APS) [66], but its effect on complement activation in the TBI brain has no reports, that needs further research.

## Conclusion

In conclusion, we observed that HCQ treatment notably alleviated microglia-induced neuroinflammation and immune cell infiltration, and the possible mechanisms, at least in part, involved the TLR4/NF-κB signaling pathway. In addition, HCQ markedly attenuated BBB disruption and brain edema by upregulating tight junction protein expression.

## Supplementary Information

**Additional file 1. Fig. S1. **HCQ downregulated the protein level of TLR4, p-NF-κB p65 and NF-κB p65 in the spleen at 3d after TBI. **Fig. S2.** The effect of HCQ on TLR4 expression was concentrated in microglia at 3d after TBI.

## Data Availability

The data sets supporting the conclusions of this article are available from the corresponding author, on reasonable request.

## Abbreviations

TBI: Traumatic brain injury
CQ: Chloroquine
HCQ: Hydroxychloroquine
DAMPs: Damage-associated molecular patterns
TJ: Tight junction
BBB: Blood brain barrier
CCI: Controlled cortical impact
TLR4: Toll-like receptor 4
TLR: Toll-like receptors
IP: Intraperitoneal injection
EB: Evans blue
PBS: Phosphate buffered saline
WW: Wet weight
DW: Dry weight
BSA: Bovine serum albumin
TTC: 2,3,5-triphenyltetrazolium chloride
mNSS: Modified neurological severity score
HBSS: Hank's balanced salt solution
FBS: Fetal bovine serum

## References

[1] Sun M, McDonald SJ, Brady RD, O'Brien TJ, Shultz SR (2018). The influence of immunological stressors on traumatic brain injury. Brain Behav Immun.

[2] Van den Brand CL, Karger LB, Nijman STM, Hunink MGM, Patka P, Jellema K (2018). Traumatic brain injury in the Netherlands, trends in emergency department visits, hospitalization and mortality between 1998 and 2012. Eur J Emerg Med.

[3] Vespa P (2017). Traumatic brain injury is a longitudinal disease process. Curr Opin Neurol.

[4] Di Pietro V, Yakoub KM, Caruso G, Lazzarino G, Signoretti S, Barbey AK (2020). Antioxidant therapies in traumatic brain injury. Antioxidants-Basel.

[5] Schepici G, Silvestro S, Bramanti P, Mazzon E (2020). Traumatic brain injury and stem cells: An overview of clinical trials, the current treatments and future therapeutic approaches. Med Lith.

[6] Alam A, Thelin EP, Tajsic T, Khan DZ, Khellaf A, Patani R (2020). Cellular infiltration in traumatic brain injury. J Neuroinflamm.

[7] Minami M, Kuraishi Y, Satoh M (1991). Effects of kainic acid on messenger RNA levels of IL-1 beta, IL-6, TNF alpha and LIF in the rat brain. Biochem Biophys Res Commun.

[8] Yatsiv I, Morganti-Kossmann MC, Perez D, Dinarello CA, Novick D, Rubinstein M (2002). Elevated intracranial IL-18 in humans and mice after traumatic brain injury and evidence of neuroprotective effects of IL-18-binding protein after experimental closed head injury. J Cerebr Blood F Met.

[9] Xu X, Yin DP, Ren HL, Gao WW, Li F, Sun DD (2018). Selective NLRP3 inflammasome inhibitor reduces neuroinflammation and improves long-term neurological outcomes in a murine model of traumatic updates brain injury. Neurobiol Dis.

[10] Gao WW, Zhao ZL, Yu GJ, Zhou ZW, Zhou Y, Hu TT (2015). VEGI attenuates the inflammatory injury and disruption of blood-brain barrier partly by suppressing the TLR4/NF-kappa B signaling pathway in experimental traumatic brain injury. Brain Res.

[11] Bergold PJ (2016). Treatment of traumatic brain injury with anti-inflammatory drugs. Exp Neurol.

[12] Hellewell S, Semple BD, Morganti-Kossmann MC (2016). Therapies negating neuroinflammation after brain trauma. Brain Res.

[13] Simon DW, McGeachy MJ, Bayir H, Clark RSB, Loane DJ, Kochanek PM (2017). The far-reaching scope of neuroinflammation after traumatic brain injury. Nat Rev Neurol.

[14] Corrigan F, Mander KA, Leonard AV, Vink R (2016). Neurogenic inflammation after traumatic brain injury and its potentiation of classical inflammation. J Neuroinflamm..

[15] Tao L, Li D, Liu H, Jiang F, Xu Y, Cao Y (2018). Neuroprotective effects of metformin on traumatic brain injury in rats associated with NF-kappaB and MAPK signaling pathway. Brain Res Bull.

[16] Zhang R, Liu Y, Yan K, Chen L, Chen XR, Li P (2013). Anti-inflammatory and immunomodulatory mechanisms of mesenchymal stem cell transplantation in experimental traumatic brain injury. J Neuroinflamm.

[17] Simon DW, McGeachy MJ, Bayir H, Clark RSB, Loane DJ, Kochanek PM (2017). The far-reaching scope of neuroinflammation after traumatic brain injury. Nat Rev Neurol.

[18] Sun P, Zhang K, Hassan SH, Tang XL, Pu HJ, Liu K (2020). Endothelium-targeted deletion of microRNA-15a/16-1 cluster promotes post-stroke angiogenesis and improves long-term neurological functions. Circ Res.

[19] Chen X, Wu S, Chen C, Xie B, Fang Z, Hu W (2017). Omega-3 polyunsaturated fatty acid supplementation attenuates microglial-induced inflammation by inhibiting the HMGB1/TLR4/NF-kappaB pathway following experimental traumatic brain injury. J Neuroinflamm.

[20] Kumar A, Stoica BA, Loane DJ, Yang M, Abulwerdi G, Khan N (2017). Microglial-derived microparticles mediate neuroinflammation after traumatic brain injury. J Neuroinflamm.

[21] Bahadoram M, Keikhaei B, Saeedi-Boroujeni A, Mahmoudian-Sani MR (2021). Chloroquine/hydroxychloroquine: an inflammasome inhibitor in severe COVID-19?. Naunyn Schmiedeberg' Arch Pharmacol..

[22] Fanouriakis A, Kostopoulou M, Alunno A, Aringer M, Bajema I, Boletis JN (2019). 2019 update of the EULAR recommendations for the management of systemic lupus erythematosus. Ann Rheum Dis.

[23] Shee JC (1953). Lupus erythematosus treated with chloroquine. Lancet.

[24] Gomez-Guzman M, Jimenez R, Romero M, Sanchez M, Zarzuelo MJ, Gomez-Morales M (2014). Chronic hydroxychloroquine improves endothelial dysfunction and protects kidney in a mouse model of systemic lupus erythematosus. Hypertension.

[25] Tang TT, Lv LL, Pan MM, Wen Y, Wang B, Li ZL (2018). Hydroxychloroquine attenuates renal ischemia/reperfusion injury by inhibiting cathepsin mediated NLRP3 inflammasome activation. Cell Death Dis.

[26] Ben-Zvi I, Kivity S, Langevitz P, Shoenfeld Y (2012). Hydroxychloroquine: from malaria to autoimmunity. Clin Rev Allerg Immu.

[27] Chatre C, Roubille F, Vernhet H, Jorgensen C, Pers YM (2018). Cardiac complications attributed to chloroquine and hydroxychloroquine: a systematic review of the literature. Drug Saf.

[28] Koch MW, Zabad R, Giuliani F, Hader W, Lewkonia R, Metz L (2015). Hydroxychloroquine reduces microglial activity and attenuates experimental autoimmune encephalomyelitis. J Neurol Sci.

[29] Kuznik A, Bencina M, Svajger U, Jeras M, Rozman B, Jerala R (2011). Mechanism of endosomal TLR inhibition by antimalarial drugs and imidazoquinolines. J Immunol.

[30] Torigoe M, Sakata K, Ishii A, Iwata S, Nakayamada S, Tanaka Y (2018). Hydroxychloroquine efficiently suppresses inflammatory responses of human class-switched memory B cells via Toll-like receptor 9 inhibition. Clin Immunol.

[31] Cui CM, Gao JL, Sun Y, Sun LQ, Wang YC, Wang KJ (2015). Chloroquine exerts neuroprotection following traumatic brain injury via suppression of inflammation and neuronal autophagic death. Mol Med Repm.

[32] Chen J, Hu J, Liu H, Xiong Y, Zou YC, Huang WT (2018). FGF21 protects the blood-brain barrier by upregulating PPAR gamma via FGFR1/beta-klotho after traumatic brain injury. J Neurotraum.

[33] Wang X, Zhu LY, Hu J, Guo RL, Ye SS, Liu F (2020). FGF21 Attenuated LPS-induced depressive-like behavior via inhibiting the inflammatory pathway. Front Pharmacol.

[34] Guan FX, Huang TJ, Wang XX, Xing Q, Gumpper K, Li P (2019). The TRIM protein Mitsugumin 53 enhances survival and therapeutic efficacy of stem cells in murine traumatic brain injury. Stem Cell Res Ther.

[35] Cui X, Chopp M, Shehadah A, Zacharek A, Kuzmin-Nichols N, Sanberg CD (2012). Therapeutic benefit of treatment of stroke with simvastatin and human umbilical cord blood cells: neurogenesis, synaptic plasticity, and axon growth. Cell Transplant.

[36] Chen J, Wang X, Hu J, Du JT, Dordoe C, Zhou QL (2021). FGF20 protected against BBB disruption after traumatic brain injury by upregulating junction protein expression and inhibiting the inflammatory response. Front Pharmacol..

[37] Liu FY, Cai J, Wang C, Ruan W, Guan GP, Pan HZ (2018). Fluoxetine attenuates neuroinflammation in early brain injury after subarachnoid hemorrhage: a possible role for the regulation of TLR4/MyD88/NF-B signaling pathway. J Neuroinflamm.

[38] Wang DX, Liu F, Zhu LY, Lin P, Han FY, Wang X (2020). FGF21 alleviates neuroinflammation following ischemic stroke by modulating the temporal and spatial dynamics of microglia/macrophages. J Neuroinflamm.

[39] Paudel YN, Shaikh MF, Chakraborti A, Kumari Y, Aledo-Serrano A, Aleksovska K (2018). HMGB1: a common biomarker and potential target for TBI, neuroinflammation, epilepsy, and cognitive dysfunction. Front Neurosci.

[40] Das M, Mohapatra S, Mohapatra SS (2012). New perspectives on central and peripheral immune responses to acute traumatic brain injury. J Neuroinflamm.

[41] Jolivel V, Bicker F, Biname F, Ploen R, Keller S, Gollan R (2015). Perivascular microglia promote blood vessel disintegration in the ischemic penumbra. Acta Neuropathol.

[42] Wu F, Chen Z, Tang C, Zhang J, Cheng L, Zuo H (2017). Acid fibroblast growth factor preserves blood-brain barrier integrity by activating the PI3K-Akt-Rac1 pathway and inhibiting RhoA following traumatic brain injury. Am J Transl Res.

[43] Sharma R, Kambhampati SP, Zhang Z, Sharma A, Chen S, Duh EI (2020). Dendrimer mediated targeted delivery of sinomenine for the treatment of acute neuroinflammation in traumatic brain injury. J Control Release.

[44] Scherbel U, Raghupathi R, Nakamura M, Saatman KE, Trojanowski JQ, Neugebauer E (1999). Differential acute and chronic responses of tumor necrosis factor-deficient mice to experimental brain injury. P Natl Acad Sci USA.

[45] Stahel PF, Shohami E, Younis FM, Kariya K, Otto VI, Lenzlinger PM (2000). Experimental closed head injury: analysis of neurological outcome, blood-brain barrier dysfunction, intracranial neutrophil infiltration, and neuronal cell death in mice deficient in genes for pro-inflammatory cytokines. J Cereb Blood Flow Metab.

[46] Goldman FD, Gilman AL, Hollenback C, Kato RM, Premack BA, Rawlings DJ (2000). Hydroxychloroquine inhibits calcium signals in T cells: a new mechanism to explain its immunomodulatory properties. Blood.

[47] Tang J, Kang Y, Huang L, Feng X, Wu L, Peng Y (2021). Neuroprotective effects of Hemocoagulase Agkistrodon on experimental traumatic brain injury. Brain Res Bull.

[48] Rosenberg GA, Yang Y (2007). Vasogenic edema due to tight junction disruption by matrix metalloproteinases in cerebral ischemia. Neurosurg Focus.

[49] Zhai K, Duan H, Wang W, Zhao S, Khan GJ, Wang M (2021). Ginsenoside Rg1 ameliorates blood-brain barrier disruption and traumatic brain injury attenuating macrophages derived exosomes miR-21 release. Acta Pharm Sin B.

[50] Yin S, Xia C, Wang Y, Wan D, Rao J, Tang X (2018). Dual receptor recognizing liposomes containing paclitaxel and hydroxychloroquine for primary and metastatic melanoma treatment via autophagy-dependent and independent pathways. J Control Release.

[51] Chou KY, Chen PC, Chang AC, Tsai TF, Chen HE, Ho CY (2021). Attenuation of chloroquine and hydroxychloroquine on the invasive potential of bladder cancer through targeting matrix metalloproteinase 2 expression. Environ Toxicol.

[52] Tsuji G, Takai-Yumine A, Kato T, Furue M (2021). Metalloproteinase 1 downregulation in neurofibromatosis 1: therapeutic potential of antimalarial hydroxychloroquine and chloroquine. Cell Death Dis.

[53] Leitner GR, Wenzel TJ, Marshall N, Gates EJ, Klegeris A (2019). Targeting toll-like receptor 4 to modulate neuroinflammation in central nervous system disorders. Expert Opin Ther Tar.

[54] Zusso M, Lunardi V, Franceschini D, Pagetta A, Lo R, Stifani S (2019). Ciprofloxacin and levofloxacin attenuate microglia inflammatory response via TLR4/NF-kB pathway. J Neuroinflamm.

[55] Jiang H, Wang Y, Liang X, Xing X, Xu X, Zhou C (2018). Toll-like receptor 4 knockdown attenuates brain damage and neuroinflammation after traumatic brain injury via inhibiting neuronal autophagy and astrocyte activation. Cell Mol Neurobiol.

[56] Sun ZZ, Nyanzu M, Yang S, Zhu XH, Wang KK, Ru JN (2020). VX765 attenuates pyroptosis and HMGB1/TLR4/NF-kappa B pathways to improve functional outcomes in TBI mice. Oxid Med Cell Longev.

[57] Marchetti T, Ruffatti A, Wuillemin C, De Moerloose P, Cohen M (2014). Hydroxychloroquine restores trophoblast fusion affected by antiphospholipid antibodies. J Thromb Haemost.

[58] Yasuda H, Leelahavanichkul A, Tsunoda S, Dear JW, Takahashi Y, Ito S (2008). Chloroquine and inhibition of Toll-like receptor 9 protect from sepsis-induced acute kidney injury. Am J Physiol-Renal.

[59] Xu R, Ji Z, Xu C, Zhu J (2018). The clinical value of using chloroquine or hydroxychloroquine as autophagy inhibitors in the treatment of cancers: a systematic review and meta-analysis. Medicine (Baltimore).

[60] Liu LQ, Wang SB, Shao YF, Shi JN, Wang W, Chen WY (2019). Hydroxychloroquine potentiates the anti-cancer effect of bevacizumab on glioblastoma via the inhibition of autophagy. Biomed Pharmacother..

[61] Zhang JY, Peng C, Shi H, Wang S, Wang Q, Wang JZ (2009). Inhibition of autophagy causes tau proteolysis by activating calpain in rat brain. J Alzheimers Dis.

[62] Liu C, Gao Y, Barrett J, Hu B (2010). Autophagy and protein aggre- gation after brain ischemia. J Neurochem.

[63] Hammad A, Westacott L, Zaben M (2018). The role of the complement system in traumatic brain injury: a review. J Neuroinflammation.

[64] Kaczorowski SL, Schiding JK, Toth CA, Kochanek PM (1995). Effect of soluble complement receptor-1 on neutrophil accumulation after traumatic brain injury in rats. J Cereb Blood Flow Metab.

[65] Ruseva MM, Ramaglia V, Morgan BP, Harris CL (2015). An anticomplement agent that homes to the damaged brain and promotes recovery after traumatic brain injury in mice. Proc Natl Acad Sci U S A.

[66] Bertolaccini ML, Contento G, Lennen R, Sanna G, Blower PJ, Ma MT (2016). Complement inhibition by hydroxychloroquine prevents placental and fetal brain abnormalities in antiphospholipid syndrome. J Autoimmun.

