# Supplementary material for: Hydroxychloroquine attenuates neuroinflammation following traumatic brain injury by regulating the TLR4/NF-κB signaling pathway
Source: J Neuroinflammation. 2022 Mar 28;19:71. doi: 10.1186/s12974-022-02430-0 (PMC8961949; doi:10.1186/s12974-022-02430-0)
Supplement: Supplementary file 1 — Additional file 1. Fig. S1. HCQ downregulated the protein level of TLR4, p-NF-κB p65 and NF-κB p65 in the spleen at 3d after TBI. Fig. S2. The effect of HCQ on TLR4 expression was concentrated in microglia at 3d after TBI. [file 12974_2022_2430_MOESM1_ESM.docx]

Additional file 1

Hydroxychloroquine attenuates neuroinflammation following traumatic brain injury by regulating the TLR4/NF-κB signaling pathway

Jian Hu^1,2^, Xue Wang^2^, Xiongjian Chen^2^, Yani Fang^2^, Kun Chen^2^, Wenshou Peng^1,2^, Zhengyi Wang^2^, Kaiming Guo^2^, Xianxi Tan^1^, Li Lin^1,2,3*^, Ye Xiong^1*^

^1^The First Affiliated Hospital of Wenzhou Medical University, Wenzhou, 325000, China

^2^School of Pharmaceutical Sciences, Wenzhou Medical University, Wenzhou, 325035, China

^3^Research Units of Clinical Translation of Cell Growth Factors and Diseases Research, Chinese Academy of Medical Science, Beijing, 100730, China

*** Correspondence:**

Li Lin

linliwz@163.com

School of Pharmaceutical Sciences, Wenzhou Medical University, University-town, Wenzhou, 325035, China

Ye Xiong

xiongye2310@163.com

The First Affiliated Hospital of Wenzhou Medical University, Wenzhou, 325000, China


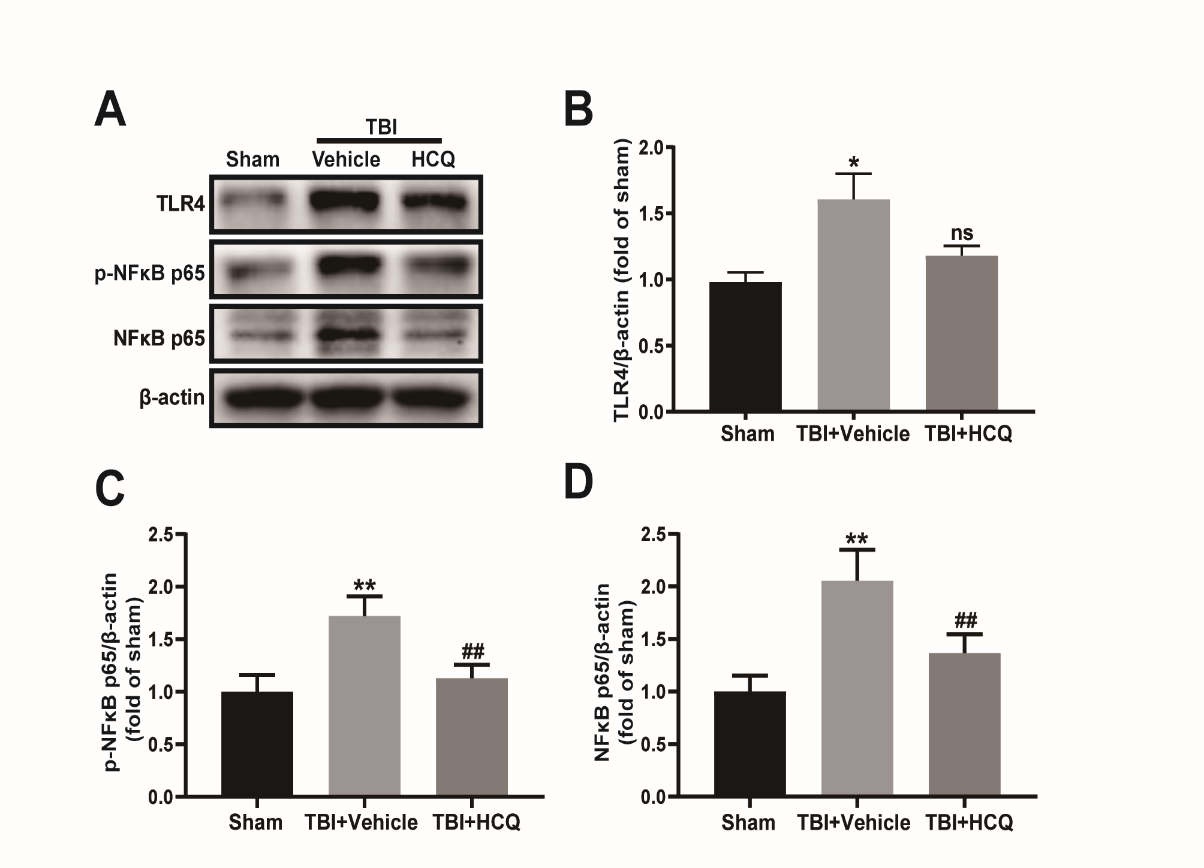


**Fig. S1** HCQ downregulated the protein level of TLR4, p-NF-κB p65 and NF-κB p65 in the spleen at 3d after TBI. **a** Representative western blot bands 0f TLR4, p-NF-κB p65 and NF-κB p65. **b-d** Densitometric quantification of TLR4, p-NF-κB p65 and NF-κB p65. n = 5 per group. The data are presented as the mean±SD. ^*^*p* < 0.05, ^**^*p* < 0.01 vs. sham group. ^##^*p* < 0.01 vs. TBI+Vehicle group. ns: no significant difference.


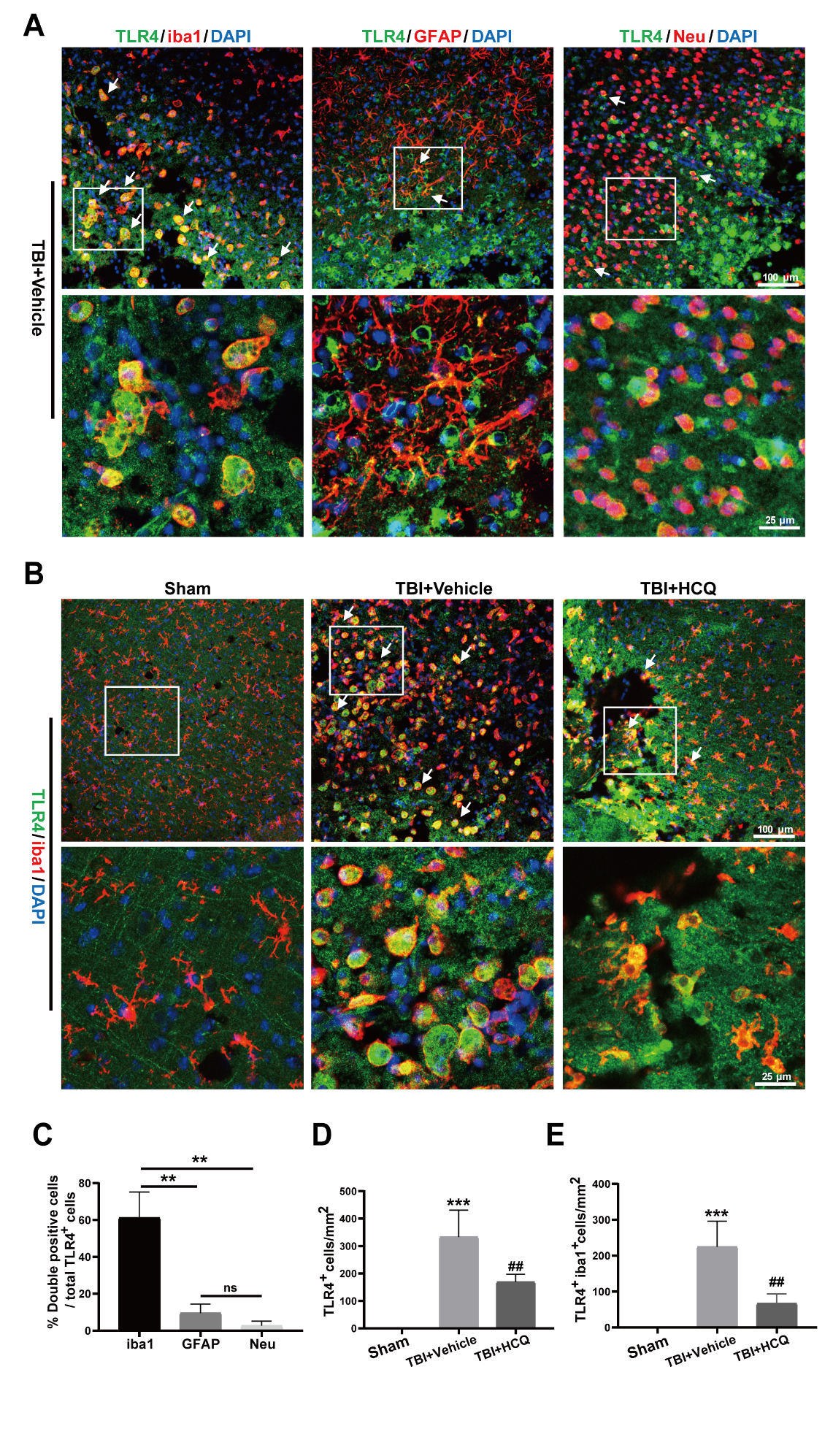


**Fig. S2** The effect of HCQ on TLR4 expression was concentrated in microglia at 3d after TBI. **a** Immunofluorescent co-staining of TLR4 (green) with iba1 (red), GFAP (red) or Neu (red). **b** Immunofluorescent staining of TLR4(green) and iba1 (red) in sham, TBI + Vehicle and TBI + HCQ group. **c** The quantification of TLR4^+^ iba1^+^, TLR4^+^ GFAP^+^ and TLR4^+^ Neu^+^cells. n = 5 per group. The data are presented as the mean±SD. ^**^*p* < 0.01 vs. iba1group. ns: no significant difference. **d,e** The quantification of TLR4^+^, TLR4^+^ iba1^+^ cells. n = 5 per group. The data are presented as the mean±SD. ^***^*p* < 0.001 vs. sham group. ^##^*p* < 0.01 vs. TBI+Vehicle group.
